# Preclinical development and clinical safety assessment of a synthetic peptide conjugate enabling endogenous antibody binding to promote innate receptor engagement

**DOI:** 10.1016/j.omton.2025.200954

**Published:** 2025-02-20

**Authors:** Erika A.K. Fletcher, Robert A. Cordfunke, Aikaterini Nasi, Gunilla Törnqvist, Rob R.P.M. Valentijn, Anna Bergqvist, Marita Westhrin, Wenche Rasch, Frida Lindqvist, Inken Dillmann, Martin Lord, Neanke Bouwman, Jacques J. Neefjes, Kees L.M.C. Franken, Stephanie McArdle, Murrium Ahmad, Silvia Johansson, Ferry Ossendorp, Michael Haggman, Maria Lampinen, Gustav Ullenhag, Sam Ladjevardi, Justyna Leja-Jarblad, Wolfgang Lilleby, Jan Wouter Drijfhout, Sara M. Mangsbo

**Affiliations:** 1 Department of Pharmacy, Science for Life Laboratory, Uppsala University, 751 24 Uppsala, Sweden; 2 Immuneed AB, 753 41 Uppsala, Sweden; 3 Department of Immunology, Leiden University Medical Center, 2300 Leiden, the Netherlands; 4 Ultimovacs ASA, 0379 Oslo, Norway; 5 Department of Clinical Pharmacy and Toxicology, Leiden University Medical Center, 2300 Leiden, the Netherlands; 6 Department of Cell and Chemical Biology, Leiden University Medical Center, 2333 Leiden, the Netherlands; 7 Department of Infectious Diseases, Leiden University Medical Center, 2300 Leiden, the Netherlands; 8 Nottingham Trent University, Nottingham NG1 4FQ, UK; 9 Department of Immunology, Genetics and Pathology, Science for Life Laboratory, Uppsala University, 751 85 Uppsala, Sweden; 10 Uppsala Akademiska sjukhuset, 751 85 Uppsala, Sweden; 11 Department of Surgical Sciences, Uppsala University, 751 85 Uppsala, Sweden; 12 Oslo University Hospital, 0450 Oslo, Norway

**Keywords:** MT: Regular Issue, prostate cancer, peptide-conjugate vaccine, tetanus toxoid, immunotherapy, dendritic cells, cancer vaccine

## Abstract

Peptide-based vaccines can be used to deliver tumor-specific antigens to dendritic cells (DCs), leading to tumor-directed T cell responses. We previously developed a peptide-peptide conjugate technology enabling *in vivo* cross-linking of pre-existing tetanus toxin-directed antibodies, facilitating antigen delivery to, and activation of DCs. To achieve this, multiple identical tetanus toxin-derived B cell epitopes (MTTEs) are conjugated to synthetically produce target antigens of choice. Herein, we describe the generation of a prostate cancer vaccine candidate (TENDU) based on this technology. It includes long synthetic peptides harboring epitopes (CD4 and CD8) from prostate-specific antigen (PAP) and prostate-specific membrane antigen (PSMA). The preclinical efficacy of TENDU was assessed in experimental systems, and safety was evaluated in a rabbit toxicity study and a human whole blood loop assay. We also report the first clinical safety assessment of TENDU. Experimental studies showed that prostate cancer patients mounted anti-MTTE antibodies in response to tetanus vaccination with recall T cell responses detected in two patients. Transgenic humanized HLA-DR4 mice displayed T cell responses and increased anti-MTTE IgG levels after vaccination with a peptide construct including an HLA-DR4 epitope. The vaccine candidate was found safe, and a positive correlation between T cell responses and anti-MTTE antibodies was noted in the first-in-human study.

## Graphical abstract

**Figure:**
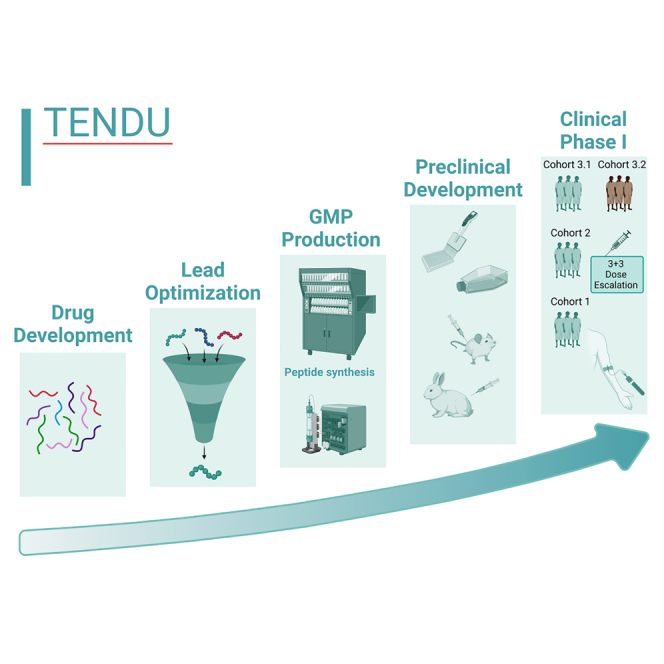

## Teaser

Fletcher and colleagues present TENDU, a prostate cancer vaccine leveraging tetanus toxin antibodies to enhance antigen delivery to dendritic cells. Preclinical studies demonstrated GMP production feasibility, safety, and efficacy in mice, rabbits, and a human *ex vivo* system. A first-in-human trial confirmed safety and showed promising immune responses.

## Introduction

Prostate cancer is the second most common malignancy in men worldwide and a leading cause of cancer deaths.^1^ Treatment options for patients with low- to intermediate-risk prostate cancer typically involve radical prostatectomy or radiation therapy, whereas high-risk prostate cancer is usually treated with androgen deprivation therapy (ADT) in combination with radiotherapy; metastatic disease is treated with ADT and chemotherapy.^2^ However, in the metastatic setting, ADT only provides a transient therapeutic benefit, and the majority of the patients will eventually progress to castration-resistant prostate cancer with limited treatment options.^3^^,^^4^ The development of novel treatment strategies is therefore essential for this group of patients. One promising approach is immunotherapy, including immune checkpoint inhibitors and other strategies to boost the immune system. Prostate cancer antigens are well described in the literature and different vaccination designs have been developed for the treatment of prostate cancer patients, including the cell-based vaccine sipuleucel-T (Provenge), which targets prostatic acid phosphatase (PAP).^5^^,^^6^^,^^7^^,^^8^ As the prostate is a non-essential organ, the target of a prostate cancer vaccine does not need to be tumor-specific, instead it can be directed to the prostate tissue itself. The best known target antigens include the above-mentioned PAP, as well as prostate-specific antigen (PSA), prostate-specific membrane antigen (PSMA), and prostate stem cell antigen (PSCA).^9^

An alternative to a cell-based vaccine is a peptide-based vaccine that can be used to load antigen-presenting cells (APCs) *in vivo* and thereby perform T cell activation within the body. Synthetic long peptides (SLPs) require uptake and processing by dendritic cells (DCs), and they can harbor multiple CD4+ and CD8+ epitopes, enabling a durable immune response.^10^ A peptide-based vaccine should include antigens derived from known proteins expressed by prostate cancer. Preferably, longer peptides can be used to enable the presentation of multiple HLA epitopes, thereby avoiding too stringent patient selection criteria. Such a vaccine should also include an adjuvant that can trigger activation of DCs, along with a method to ensure that peptides are taken up by the same DC *in vivo* post-injection of the drug.

Various methods to enhance DC uptake and antigen presentation have been evaluated for cancer therapy, including *ex vivo*-generated DCs pulsed with tumor antigens in combination with cytokines or toll-like receptor (TLR) agonists, DCs transfected (virally or biochemically) with a genetic vector coding for tumor antigens and immunostimulatory factors, or DC-derived exosomes.^11^^,^^12^ The development of mRNA vaccines during the COVID-19 pandemic paved the way for new strategies in cancer treatment. Lipid nanoparticles (LNPs) are employed to protect mRNA from degradation and to enhance its intracellular delivery. To further optimize the therapeutic efficacy of mRNA, site-specific LNPs have been developed, ensuring selective targeting of organs, tissues, or cells. This can be achieved by adjusting the negative net charge of the LNP, by adjusting the proportions of lipid components, or by coating the LNP with specific antibodies.^13^^,^^14^^,^^15^

We have previously developed a strategy facilitating antigen delivery to DCs, as well as inducing DC activation, using a peptide-peptide conjugate technology. The synthetic peptide-peptide conjugate preparation ensures that antigen uptake and activation take place in the same DC in order to obtain adequate T cell activation. The rationale behind our strategy is that by ensuring immune-complex formation *in vivo*, we also ensure immune activation and facilitation of DC cross-presentation.^16^ Most people have endogenous antibodies against tetanus toxoid (TTd) due to national vaccination programs. The pre-existing anti-tetanus toxoid antibodies in circulation thus allow for immune-complex formation, which can be used to drive immunogenicity directed to other parts of the peptide-peptide conjugate. We have earlier reported the finding of an unstructured B cell epitope derived from tetanus toxin origin (referred to as MTTE from here on) and developed a peptide conjugate technique enabling the conjugation of multiple identical copies of MTTE covalently attached to one or more T cell epitopes of choice.^17^ The resulting immune complexes formed via the conjugate strategy improved antigen uptake by DCs and consequently improved CD8+ T cell recall responses in a human *ex vivo* blood loop system.^18^

Herein, we describe the generation of a prostate cancer vaccine candidate (TENDU) based on our MTTE-conjugate technology aiming to improve peptide immunogenicity via immune-complex formation *in vivo*. TENDU was designed to include long synthetic peptides harboring prostate cancer-derived epitopes (CD4 and CD8) from PAP and PSMA, as well as the previously identified prostate cancer antigen NY-ESO1.^19^ The preclinical efficacy of the vaccine candidate was assessed in experimental systems, and a rabbit toxicity study as well as a whole blood loop assay were used to evaluate the safety of the construct. We also report the first clinical safety assessment of the vaccine platformed in the trial TENDU101 [ClinicalTrials.gov ID: NCT04701021].

## Results

### Optimization of the conjugation core element

**Figure 1:**
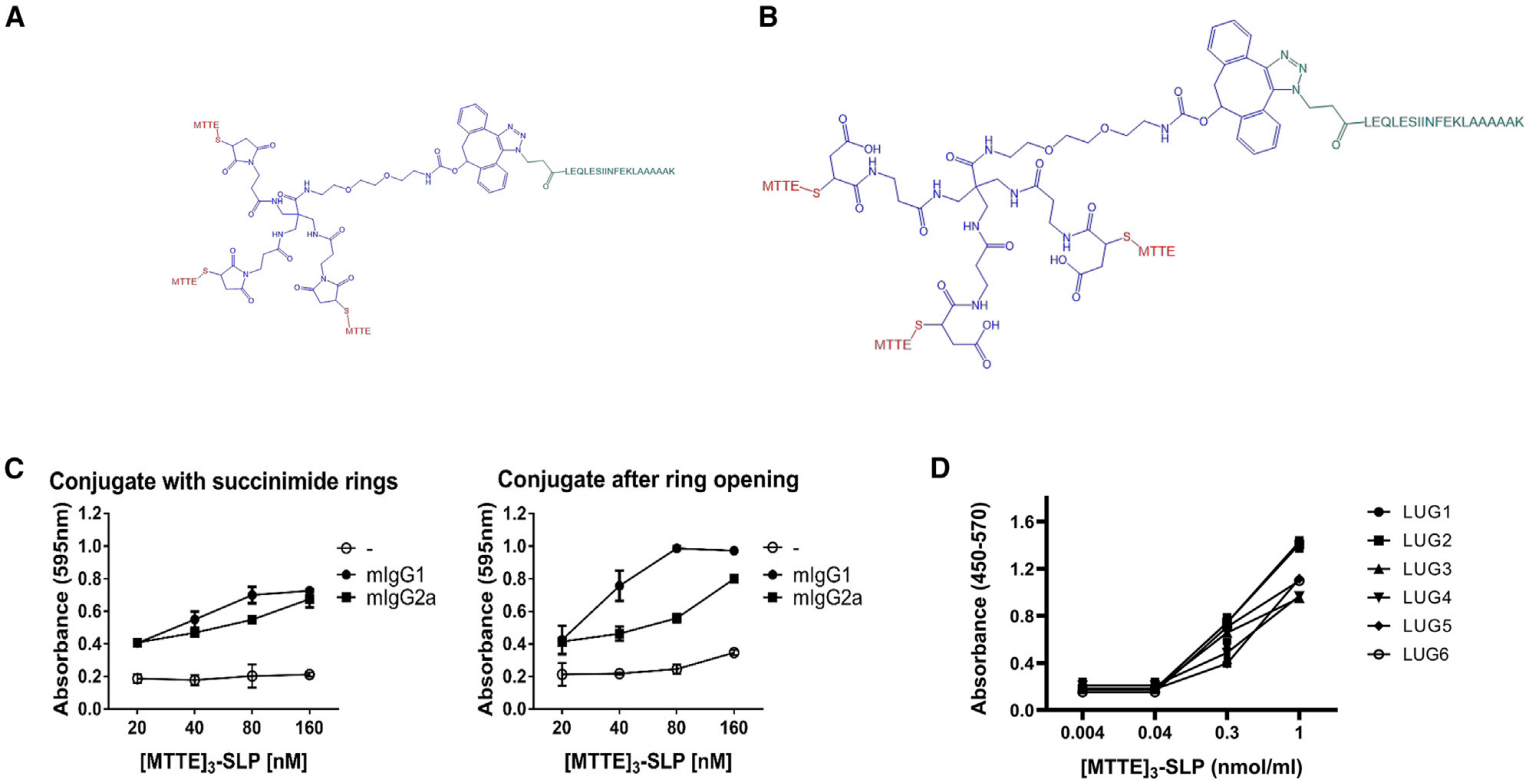
Maintained function of MTTE-peptide conjugates after succinimide ring opening MTTE-peptide conjugates ([MTTE]_3_-SLP) were synthesized and are illustrated either with (A) succinimide rings or (B) after ring opening, both variants with an OVA-derived CD8+ epitope (LEQLESIINFEKLAAAAAK) in the SLP peptide. Red: MTTE, blue: core structure, green: SLP. (C) The biological effect of [MTTE]_3_- SIINFEKL conjugates, comparing intact and opened succinimide rings, was measured as β-galactosidase activity of SIINFEKL-specific T cells (B3Z cells) co-cultured with dendritic cells and activity was assessed by the level of substrate cleavage at 595nm. mIgG1 = mouse IgG1; mIgG2a = mouse IgG2a; MTTE = minimal tetanus toxin epitope; SLP = synthetic long peptide. Results are shown as mean ± SD. (D) Maintained binding of GMP LUG-1-6 constructs to recombinant anti-MTTE IgG1 antibody as assessed by ELISA.

In the context of GMP production of peptide-peptide conjugates, we evaluated the core element that enables the conjugation of three tetanus epitopes to the SLPs. In our previous work, MTTE constructs were made to include succinimide rings. Due to reported instability of these ring structures,^20^ we assessed the stability of the constructs with succinimide rings in pH 8.7 at 30°C, at time points 0 h and 46 h. Under these conditions, virtually all succinimide rings were hydrolyzed at the 46-h time point (Figure S1). Additionally, a proportion of molecules also lost the MTTE-SH sequence. Thus, to ensure stability, an extra incubation step at pH 8.7, resulting in ring opening, was applied for future construct production. To confirm that ring opening of the constructs did not affect the biological response, constructs with both open and closed rings were made with an imbedded OVA-derived model CD8 epitope (SIINFEKL). These constructs ([MTTE]_3_-LEQLESIINFEKLAAAAAK) were incubated with murine growth-dependent D1 dendritic cells that were subsequently co-cultured with B3Z hybridoma cells, e.g., murine T cells with an SIINFEKL-specific TCR that produces B-galactosidase upon MHC-I/peptide-TCR engagement. Constructs with both open and closed succinimide rings were able to activate B3Z cells, confirming that the constructs with open rings were biologically functional (Figures 1A–1C).

### Epitope selection and peptide-peptide conjugate design for GMP-grade manufacturing

We developed a series of peptide-peptide conjugates each comprising one SLP with CD4 and CD8 epitopes derived from PSMA and PAP. For the generation of an efficient peptide vaccine for cancer, the T cell-specific epitopes need to cover a wide range of HLA alleles present in the human population due to polymorphism of HLA alleles. The selection of SLP epitopes was therefore initiated with a search in the Eurotransplant database 2013, where we identified class I HLA types that together covered 96% of a population of 4,000 donors (Table 1, i). Based on this selection, we identified by literature search^21^^,^^22^^,^^23^^,^^24^ 11 prostate cancer-specific CD8 epitopes (Table 1, ii). Prostate cancer-specific CD4 epitopes were selected based on the criteria of either promiscuously presented or presented by multiple class II alleles (Table 1, iii).^25^^,^^26^^,^^27^ To identify the optimal SLP combinations for processing and presentation by HLA molecules, all possible combinations of the 11 CD8 epitopes and the six CD4 epitopes were generated, resulting in 66 SLPs. This set of peptides was then comprehensively analyzed to determine whether this fully synthetic peptide-peptide conjugate design could effectively engage endogenous antibodies and facilitate Fc receptor targeting, uptake, and presentation of the conjugates.

**Table 1:** Selection of SLP epitopes

i	HLA class I	Frequency^a^
	A1	28%
	A2	50%
	A3	29%
	A11	10%
	A24	18%
	A31	5%
	A33	2%
	At least one of the types above	96%

ii	Code	CD8-epitope sequence	HLA class I allele
	C1	ILLWQPIPV	A2
	C2	YLPFRNCPR	A3, A11, A31, A33
	C3	LYCESVHNF	A24
	C4	GMPEGDLVY	A1
	C5	LLHETDSAV	A2
	C6	MMNDQLMFL	A2
	C7	VLAGGFFLL	A2
	C8	KVFRGNKVK	A3, A11, A31, A33
	C9	NYARTEDFF	A24
	C10	LLAVTSIPSV	A2
	C11	SLSLGFLFL	A2

iii	Code	CD4-epitope sequence	HLA class II allele
	H1	GQDLFGIWSKVYDPL	Promiscuous
	H2	TEDTMTKLRELSELS	Promiscuous
	H3	GKVFRGNKVKNAQLA	Promiscuous
	H4	TGNFSTQKVKMHIHS	DR4
	H5	NYTLRVDCTPLMYSL	DR1/DR9
	H6	RQIYVAAFTVQAAAE	DR4/DR9

i: frequency of class I HLA types; ii: prostate cancer-specific CD8 epitopes; iii: prostate cancer-specific CD4 epitopes.

a HLA frequency based on HLA-typing of 4,000 donors from Netherlands, Belgium, Germany, and Austria.

The analysis encompassed transporter associated with antigen processing (TAP) and proteasome assays, with the objective of enhancing epitope translocation to the endoplasmic reticulum (ER), processing, and presentation (illustrated in Figure S2). The processing of the SLPs by immune-proteasome was assessed by MALDI-TOF, and peptides with the correct C terminus for HLA binding were identified. A selection of the 66 processed peptides is presented in Table S1. SLPs with incorrect cleavage were resynthesized with various spacer elements between the CD8 and CD4 epitopes and re-tested for proteasome cleavage. Using the TAPREG prediction tool,^28^ we identified the sequence ARWW as a potential TAP Translocation-enhancing sequence (TTES). The N terminus of the selected CD8 was extended with ARWW, and the TAP translocation was analyzed in an *in vitro* assay (Table S2).

After the TAP analysis and second round of proteasome cleavage, five SLPs with different CD8 and CD4 sequences were selected based on a set of criteria that optimizes the vaccine for both physicochemical production and immunogenicity properties (Table 2 shows the final design of the vaccine constructs). The criteria used are as follows.

(1) Each construct should contain at least one CD8 epitope for every selected allele, with a preference for including two HLA-A2 epitopes, as HLA-A2 is the most common class I allele.
(2) The set should preferably contain multiple HLA-II promiscuous epitopes.
(3) In case of multiple options for the above criteria, the synthetic long peptides should be selected based on good solubility and purity.

**Table 2:** The final design of the TENDU vaccine with six peptide conjugates

Conjugate	The SLPs with both **CD8** and CD4 epitopes highlighted	Code
LUR1/LUG1^a^	XARWW^b^**NYARTEDFF**QQQPPP^c^*GQDLFGIWSKVYDPL*	C9 H1
LUR2/LUG2^a^	XARWW**LLHETDSAV**AAA*RQIYVAAFTVQAAAE*	C5 H6
LUR3/LUG3^a^	XARWW**SLSLGFLFL**AAA*GKVFRGNKVKNAQLA*	C11 H3
LUG4/LUG4^a^	XARWW**GMPEGDLVY***TGNFSTQKVKMHIHS*	C4 H4
LUG5/LUG5^a^	XARWW**KVFRGNKVK***NYTLRVDCTPLMYSL*	C8 H5
LUG6/LUG6^a^	XGARGPESRLLEFYLAMPFATPMEAELA	NY-ESO

a LUR and LUG are conjugates of the same peptide sequence with the difference of LUG being produced with GMP standard (LU**R** = research and LU**G** = GMP).

b TAP sequence: ARWW.

c Different SLPs have different proteasome sequences. X = azido propionic acid.

The sixth SLP contains a sequence from the known NY-ESO 1 antigen, a previously described promising immunogenic cancer antigen expressed in advanced prostate cancer.^19^ An NY-ESO1 MHC epitope dense region^29^ was chosen and the exact amino acid 79–105 was selected for solubility purposes. The six SLPs were conjugated to three MTTE sequences and subsequently assessed for large-scale production.

### Preclinical studies of the efficacy of prostate cancer peptide-peptide vaccine conjugates

Six distinct peptide-peptide conjugates were thus selected, each containing confirmed epitopes from PAP and PSMA, with the goal of inducing specific T cell responses targeting prostate cancer. These vaccine constructs, referred to as TENDU, were manufactured at GMP grade without loss of binding capability to anti-tetanus antibodies (Figure 1D).

#### Increased anti-MTTE antibody titers in human plasma after tetanus toxoid vaccination

**Figure 2:**
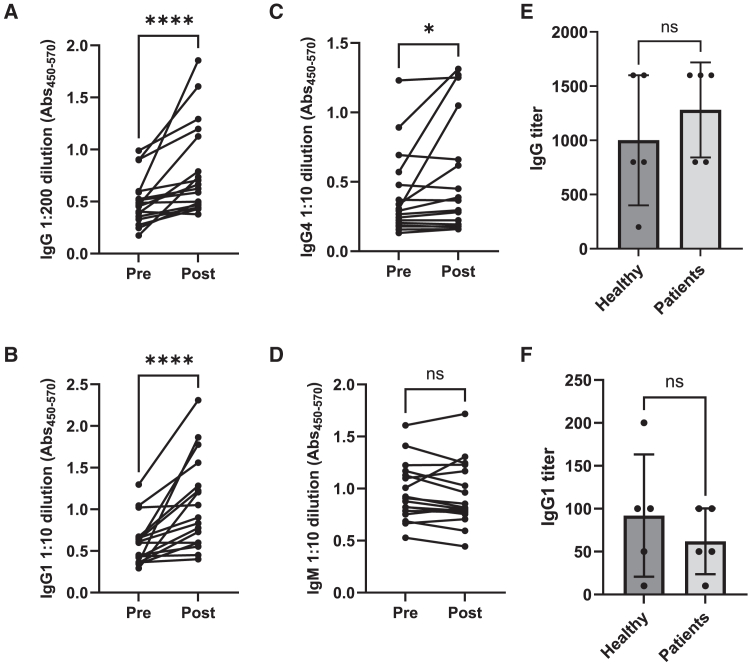
Anti-MTTE antibody titers in plasma from prostate cancer patients and healthy donors pre- and post-DTP vaccination Blood samples were collected from prostate cancer patients from the urology department and the oncology department and from healthy donors before and approximately 2 weeks after a DTP vaccination. Anti-MTTE antibody levels in plasma were determined using an in-house ELISA. The antibody titers are expressed by the dilution whereby the absorbance of MTTE is divided to the respective ETTM background signal. Levels of IgG (A), IgG1 (B), IgG4 (C), and IgM (D) in prostate cancer patients (*n* = 17) before and after DTP vaccination presented as absorbance (450–570 nm) at dilution 1:200 for IgG and 1:10 of plasma for IgG1, IgG4, and IgM. Anti-MTTE antibody titers post vaccination were analyzed in age-matched male healthy volunteers (*n* = 5) and prostate cancer patients (*n* = 5): (E) IgG titers and (F) IgG1 titers shown as median with 95% confidence interval. Antibody titers in (E) and (F) were determined based on how much the samples could be diluted before the antibodies could no longer be detected. A cutoff value for detection was set to optical absorbance = 0.100. Significance was tested with Wilcoxon matched-pairs test (A)–(D) or unpaired t test with Welch’s correction, (E) and (F); ∗∗*p* < 0.01; ∗∗∗*p* < 0.001.

Since the presence of anti-MTTE antibodies will be important for immune-complex formation, uptake of conjugates and subsequent T cell activation, anti-MTTE levels were analyzed in individuals with prostate cancer and healthy controls. Levels of immunoglobulin (Ig)G, IgG1, IgG4, and IgM were evaluated pre and post a TTd-containing vaccination in prostate cancer patients to confirm that they mount anti-MTTE antibodies similar to healthy volunteers as reported in Fletcher et al.^18^ Prostate cancer patients displayed significantly increased levels of IgG, IgG1, and IgG4, but not IgM after the TTd vaccination (Figures 2A–2D). An additional cohort of age-matched male healthy volunteers (*n* = 5) and prostate cancer patients (*n* = 5) were recruited for the preclinical safety assessment (see below); anti-MTTE-levels (total IgG and IgG1) post-TTd vaccination were similar between the two groups (Figures 2E and 2F). The levels of IgG2 and IgG3 have previously been undetectable against MTTE^18^ after tetanus vaccination in healthy individuals and were therefore not analyzed. These data indicate that even though some of the prostate cancer patients may have pre-existing anti-MTTE levels without a boost vaccination, a boost vaccination is expected to increase anti-MTTE levels in patients, as in healthy volunteers.

#### CD4^+^ and CD8^+^ T cell recall responses with LUR1-6 in DTP-vaccinated prostate cancer patients and healthy individuals

**Figure 3:**
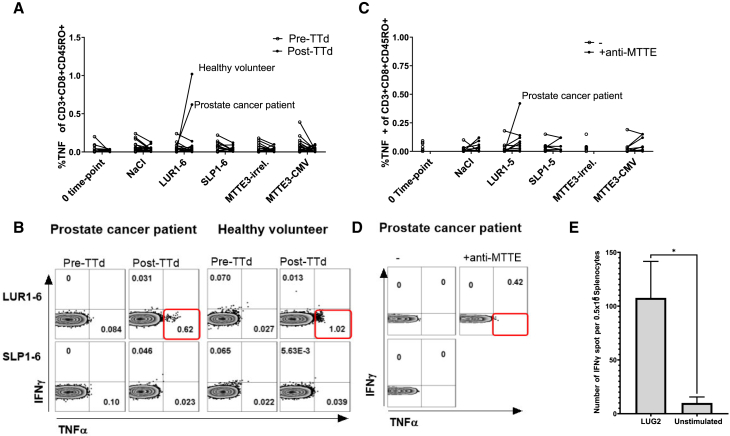
IFNγ and TNFα-release by memory T cells in response to LUR1-6 conjugates Peptides and LUR1-6 conjugates were incubated in human whole blood from prostate cancer patients and healthy donors, pre and post a DTP vaccination, (A) and (B), or with and without a mouse anti-MTTE IgG2a antibody, (C) and (D), in a circulating blood loop assay. T cell surface markers and intracellular IFN-γ and TNF-α were analyzed with flow cytometry. The conjugates tested were LUR1-6, LUG1-6 (see Table 2), and SLP1-6, which are the free synthetic long peptides of respective conjugates. The [MTTE]_3_-CMV conjugate contains the HLA-A∗0201-restricted epitope pp65(NLV) from CMV and CMV lysates were used as positive controls. MTTE3-irrelevant (MTTE-irrel.) contains a scrambled SLP sequence (DGLQGLLLGLRQRIETLEGK) without any know human T cell epitopes. Representative flow cytometry dot plots of the three responding donors from (A) and (C) are displayed in (B) and (D). The cells were gated as CD45RO + CD3+CD4-CD8+ and the % of IFN-γ+ and TNF-α+ cells are displayed. (E) SLP-specific IFN-γ producing T cells upon LUG2 vaccination in seropositive HLA-DR4 humanized transgenic mice were measured in a recall-ELISpot assay. Result shown as mean ± SD. Significance was tested with Mann Whitney-test ∗*p* < 0.05.

Research-grade TENDU constructs (LUR1-6) were tested in patient blood in the *ex vivo* blood loop assay pre and post a TTD-containing vaccination to determine if CD8+ or CD4+ T cell recall responses can be induced against the cancer epitopes in TENDU. We have previously demonstrated that a model MTTE-construct containing the CMV-derived CD8+ epitope NLVPMVATV (referred to as [MTTE]_3_-NLV) stimulates interferon (IFN)-γ and tumor necrosis factor (TNF)-α release from memory NLV-specific CD8+ T cells post 6 h stimulation in an *ex vivo* human blood loop assay.^18^ However, pre-existing T cell populations enabling direct readout were scarce in the present study with no tetramer staining available to improve sensitivity, in contrast to our previous study. Nevertheless, the mixture of LUR1-6 constructs induced TNF-α release by CD8+CD45RO + T memory cells in blood post-TTd vaccination from one patient and one healthy volunteer (Figures 3A and 3B). In blood from a non-vaccinated oncology patient, mouse anti-MTTE IgG2a was spiked in together with the LUR1-6 mixture resulting in induced TNF-α release by CD8+CD45RO+ T memory cells (Figures 3C and 3D), similar to what we have seen in healthy donors with the [MTTE]_3_-NLV construct.

#### Humanized HLA-DR4 mice show measurable T cell responses after LUG2 vaccination

Since the blood loop method had limited sensitivity in this setting, we tested the construct in humanized HLA-DR4 transgenic mice. Here, one of the peptide constructs that includes an HLA-DR4 epitope (LUG2) was chosen to evaluate T cell activation in the aforementioned mouse model. Animals that seroconverted after vaccination mounted measurable T cell responses against the SLP compared with unstimulated cells (Figure 3E).

### Preclinical evaluation of the safety of the peptide-peptide conjugates

Preclinical safety assessment was performed using the human blood *ex vivo* blood loop assay as well as a repeated toxicity study in rabbits. For the human *ex vivo* system, a new cohort of healthy individuals and prostate cancer patients was recruited and given a tetanus booster before study inclusion as blood donors. No broad cytokine release in response to LUG1-6 was noted in the *ex vivo* blood loop assay, as opposed to the positive reference substance alemtuzumab. The levels of the cytokines IFN-γ, interleukin (IL)-1β, IL-2, IL-6, IL-10, and TNF-α were similar to vehicle for all donors at all LUG1-6 concentrations tested. For all donors, IL-1β and IL-2 levels were below lower limit of quantification (LLOQ) in LUG1-6 samples at all concentrations tested. A slight but significant elevation of IL-8 and complement (C5a) was noted at the highest concentration of LUG1-6 (C5a, IL-6, IL-8, and TNF-α shown in Figure S3).

*In vivo* safety assessment of TENDU was performed in male rabbits pre-vaccinated with a tetanus vaccine to generate circulating anti-MTTE antibodies. Rabbits were assigned to groups based on the anti-MTTE titers after a tetanus vaccination cycle and were subsequently vaccinated four times with low, intermediate, or high dose of TENDU (60 μg, 600 μg, or 1,440 μg; total concentration of LUG1-6 combined). No clinical signs of toxicity were observed in any of the groups. The subcutaneous injections did not induce any local adverse reactions and there was no effect on body weight, food consumption, or body temperature of the rabbits in the study. Cytokine release responses to the conjugates in the plasma of rabbits using ELISA showed no increase in the concentration of IFN-γ at any of the LUG1-6 doses used over time (Figure S4A). IL-1b was undetectable in most of the samples (data not shown). A mild increase in IL-8 production was detected in some of the LUG1-6 vaccinated groups on week 15 irrespective of prior exposure to TT vaccination (Figure S4B). Plasma of lipopolysaccharide-activated rabbit blood was used as a positive control for IL-8; spiking of a known amount of IFN-γ to rabbit plasma was used as a positive control for IFN-γ in the ELISA assay. Notably, peptide-peptide conjugate exposure was evident as indicated by a measurable increase in anti-MTTE antibody levels at the highest dose level, not observed in the vehicle (Figure S4C). Interestingly, the seronegative group (that did not receive TTd prior to LUG1-6 exposure) did not display the same increase in anti-MTTE antibody levels, suggesting that previous tetanus toxoid exposure aided immunogenicity responses.

### Clinical trial assessment of safety of the peptide-peptide conjugate vaccine approach

To assess the safety profile of the peptide-peptide conjugate in a clinical context, a Phase I clinical trial was performed on high-risk prostate cancer patients with documented progressive disease after radical prostatectomy. The study design followed the conventional 3 + 3 dosing escalation approach using 40, 400, and 960 μg per dose for cohorts 1, 2, and 3 (3.1/3.2). Four of the six conjugates assessed in the preclinical workup were selected for the clinical trial, a decision based on production quality and yield. An intramuscularly administered TTd-containing vaccination was followed by administration of TENDU by subcutaneous administration. Each TENDU-conjugate was administered separately in the abdomen by four injections per administration round with a total of four doses administered every 2 weeks. In cohort 3.2, TTd vaccination and TENDU were both administered in the shoulder. All patients also completed a last safety follow-up visit 6 months after the last dose of TENDU.

The vaccine was deemed safe with a total of 52 adverse events (AEs) reported of which most were mild (63.5%) and to a high extent (88.4%) categorized as unrelated to the drug compound administered. The most frequent AEs were decreased lymphocyte counts, diarrhea, lipase increase, and anemia. A total of six treatment-related adverse drug events were reported. Three of these were mild, including transient injection site reactions in one of the patients in the highest dose group. There were also three events of increased lipase levels of severe intensity in the intermediate dose group (two patients) that were deemed possibly related to TENDU vaccination. The lipase levels normalized over time. No severe AEs were reported (Table 3).

**Table 3:** The TENDU-101 phase I study confirms safety of the peptide-peptide conjugate

	Any grade	Grade 3	Any grade	Grade 3
	Patients, n (%)		Events, n (%)	
Any AEs	12 (100)	4 (33)	52	6 (11.5)
Any TENDU related AEs	3 (25)	2 (16.7)	6 (11.5)	3 (5.8)
Lipase increase	2 (16.7)	2 (16.7)	3 (5.8)	3 (5.8)
Injection site reaction	1 (8.3)	0	3 (5.8)	0

**Any EAs unrelated to TENDU**				

Lymphocyte count decrease	9 (75)	1 (8.3)	9 (17.3)	1 (1.9)
Diarrhea/constipation	7 (58)	1 (8.3)	8 (15.4)	2 (17%)
Anemia	4 (33.3)	0	4 (7.7)	0
White blood cell decrease	3(25)	0	3 (5.8)	0
LDH increase	3(25)	0	3 (5.8)	0
COVID-19	2 (16.7)	0	2 (3.8)	0
Hypertension	2 (16.7)	0	2 (3.8)	0
Hyponatremia	2 (16.7)	0	2 (3.8)	0
Hypokalemia	1 (8.3)	0	1(1.9)	0
Bronchial infection	1 (8.3)	0	1(1.9)	0
Elective polyp excision	1 (8.3)	0	1(1.9)	0
Fatigue	1 (8.3)	0	1(1.9)	0
Hypercalcemia	1 (8.3)	0	1(1.9)	0
Hypoalbuminemia	1 (8.3)	0	1(1.9)	0
Nocturi	1 (8.3)	0	1(1.9)	0
Platelet count decrease	1 (8.3)	0	1(1.9)	0
Wound infection leg	1 (8.3)	0	1(1.9)	0

No systemic risk for infusion reactions (cytokine release syndrome) was detected by cytokine analyses. One patient in dose level group 3 had elevated levels of IFN-γ over baseline with a similar magnitude between the four time points at visit 4. As the IFN-γ levels were similar between all four time points of visit 4 (pre- and post-TENDU exposure), the IFN-γ released does not appear to be an immediate response to the TENDU vaccination and could thus be due to a common cold or other infection. For all other cytokines (IL-1β, IL-2, IL-4, IL-6, IL-8, IL-10, IL-12p70, IL-13, and TNF-α) the cytokine levels were low, similar to baseline or below the lower limit of quantification (LLOQ).

**Figure 4:**
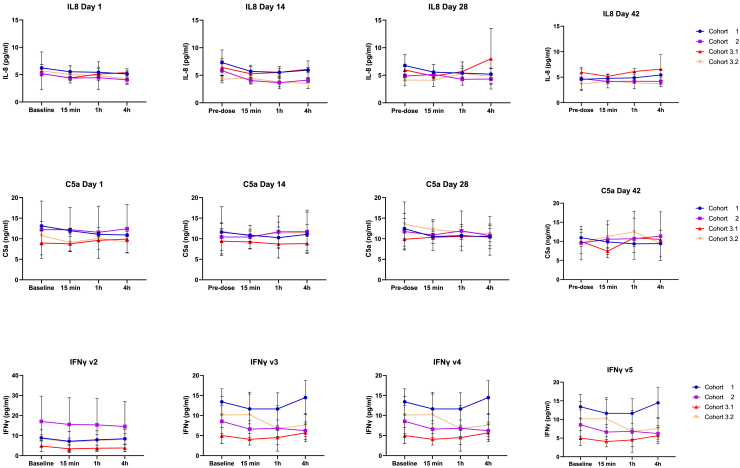
No systemic risk of cytokine release or complement activation by TENDU vaccination Plasma levels of IL-8, C5a, and IFN-γ before and after four doses of TENDU vaccination administered with a 2-week interval between each dose. At each visit, blood was collected at four time points (baseline [time 0], 15 min, 1 h, and 4 h). Cytokines were assessed using a Mesoscale V-plex kit, result expressed as pg/mL (mean values +SD). Lower and upper limit of quantifications (LLOQ and ULOQ) are verified by MSD and calculated from the standard curve and percentage recovery of diluent standards with precision of 20% and accuracy of 80%–120%. Complement activation (C5a) analyzed using ELISA kits from Hycult Biotech, results expressed as ng/mL (mean values +SD). Blue symbols (circle) represent cohort 1 (TENDU dose 40 μg); purple symbols (square) represent cohort 2 (TENDU dose 400 μg); red symbols (triangle) represent cohort 3.1 (TENDU dose 960 μg); yellow symbols (upside down triangle) represent cohort 3.2 (TENDU dose 960 μg; co-administered with tetanus toxoid in the same anatomical location).

The TENDU vaccine did not increase complement split products over baseline in any of the patients at any of the visits or time points analyzed (IL-8, C5a, and IFN-γ shown in Figure 4).

### Anti-tetanus and anti-MTTE-levels in the clinical trial samples

**Figure 5:**
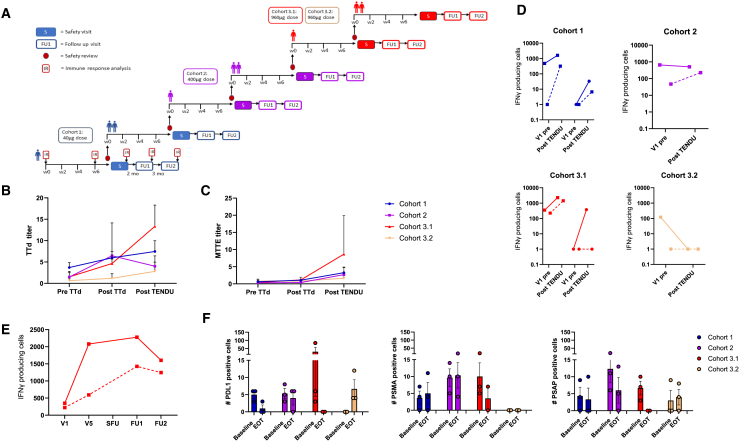
Anti-tetanus- and MTTE antibody titers, T cell responses, and preliminary anti-tumor activity after TENDU vaccination (A) Schematic diagram of the trial design. (B) Anti-tetanus (TTd) and (C) anti-MTTE antibody levels in plasma (mean ± SD) before and after tetanus toxoid-containing vaccination and after four doses of TENDU vaccination administered with a 2-week interval between each dose (*n* = 3 in each cohort). The antibody titers are expressed by the dilution whereby the absorbance of MTTE is divided to the respective ETTM background signal. (D) IFN-γ production in response to stimulation with mixes of peptides corresponding to CD4 and CD8 epitopes derived from PSA and PSMA. Each pair of lines represents one patient; solid lines show CD4-responses, dotted lines show CD8-responses. (E) T cell responses over time for the best Ab-responder patient in cohort 3.1. (F) Number of circulating tumor cells expressing PDL1, PSMA, and PSAP, respectively, at baseline and end of trial (mean ± SD, (*n* = 3 in each cohort). V1 = visit 1; EOT = end of trial; FU = follow-up. Blue symbols represent cohort 1 (TENDU dose 40 μg); purple symbols represent cohort 2 (TENDU dose 400 μg); red symbols represent cohort 3.1 (TENDU dose 960 μg); yellow symbols represent cohort 3.2 (TENDU dose 960 μg; co-administered with tetanus toxoid in the same anatomical location).

Figure 5A illustrates the study design following the conventional 3 + 3 dosing escalation approach. All patients had measurable anti-tetanus (TTd)/anti-MTTE Ab levels at baseline. Anti-tetanus levels were notably elevated throughout the study, especially in cohort 3.1. In all patients, the levels of anti-MTTE Abs increased after TENDU vaccination. The highest level of Abs specific for MTTE was measured in one patient in cohort 3.1 (the highest dose group) followed by one patient in dose group 2 and one patient in dose group 1 (collectively named the “high-MTTE group”) (Figures 5B and 5C). In cohort 3.2, the TTd vaccination did not produce the same levels of anti-TTd antibodies at visit 2 as observed in all other cohorts. This may account for the absence of anti-MTTE responses at visit 5, leading to inconclusive results for the co-administration route.

### Assessment of T cell responses

T cell responses were investigated by measuring the production of IFN-γ from T cells in response to stimulation with mixes of peptides corresponding to CD4 and CD8 epitopes derived from PAP and PSMA. An increase in the number of T cells responding to stimulation with the CD4 and/or CD8 epitopes compared with baseline was observed in 5 of 6 patients with samples from pre-and post-TENDU vaccination (Figure 5D). Interestingly, the most prominent T cell responses were noted in the patients with the highest measured anti-MTTE IgG levels after TENDU vaccination (“High-MTTE” group). The administration of TTd and TENDU to the same anatomical location (cohort 3.2), although spaced in time, did not result in improved T cell responses (Figure 5D). T cell responses over time for the best responder are shown in Figure 5E.

### Preliminary anti-tumor response

Circulating tumor cells showed a trend to be reduced in cohort 3.1 for all markers assessed (Figure 5F). No changes in blood levels of PSA and PAP levels were observed at the end of the treatment cycle of TENDU. A longer follow-up was not possible to perform because standard-of-care therapy was initiated subsequent to the end of the TENDU cycle, impacting PSA levels (data not shown).

## Discussion

Synthetic peptides are increasingly explored in cancer vaccine development for the purpose of inducing tumor-directed T cell responses.^30^ Synthetic long peptides are preferred over trimmed peptides for vaccine design as they require processing by APCs to be presented to T cells, thus reducing the risk of antigen presentation by non-APCs lacking co-stimulatory receptors.^31^ Still, formulation and delivery strategies require further development as peptides degrade rapidly *in vivo* with poor immunogenicity as a result. Critical aspects for peptide immunogenicity are the selection of the antigen(s) to be targeted and the unmet need for strong adjuvants.^32^

We have developed an adjuvant strategy by using a technology that enables cross-linking of pre-existing circulating tetanus antibodies in the body. This is achieved by employing a three-dimensional chemistry design in which multiple identical tetanus toxin-derived B cell epitopes (MTTEs) are conjugated to synthetically produced antigenic tumor peptides intended to induce T cell responses. The conjugate is designed to provide both antigen and adjuvant signals, ensuring colocalization of antigen and activation signal to the same APC.^17^^,^^18^

In the present study, we used this technology to design a prostate cancer vaccine candidate (TENDU) containing long synthetic peptides harboring prostate cancer-derived epitopes (CD4 and CD8) from PAP, PSMA (preclinical and clinical study), and NY-ESO1 (preclinical study). The conjugation of the SLPs to the MTTE-complex ensures efficient processing and cross-presentation by activated DCs, generating robust T cell responses and avoidance of tolerance.

We have previously demonstrated that the MTTE-technology can boost antigen-specific T cell responses through the formation of immune complexes in the presence of circulating antibodies.^18^ In order to improve the stability of the MTTE-complex for clinical use, we initiated work to optimize the core chemistry. Thiol-maleimide conjugation of the MTTE peptides to a core structure results in the formation of succinimide rings. The same type of chemical reaction has been extensively used for the generation of antibody-drug constructs.^33^^,^^34^ However, succinimide rings are sensitive to two types of reactions that can destabilize them, namely hydrolysis and retro-Mikael addition.^33^ We showed that vaccine constructs generated using this method have a decreased MTTE stability due to the hydrolysis of the succinimide rings. Therefore, we analyzed the capacity of open vs. closed ring constructs to induce T cell responses in the presence of anti-MTTE antibodies of different IgG isotypes. We found that the more stable constructs with open rings were equally efficient in inducing T cell activation in the presence of monoclonal anti-MTTE antibodies (mIgG1 and mIgG2). This approach was thus used from then on for all our constructs.

Next, we selected prostate-specific antigens derived from PSMA, PAP, and NY-ESO1. These proteins are known to be immunogenic, and T cells directed against epitopes derived from these antigens have been identified in the circulation of prostate cancer patients.^5^^,^^9^^,^^23^^,^^35^^,^^36^^,^^37^ One limitation of peptide vaccines is their restricted coverage of HLA alleles within the human population. Another may be the absence of multiple prostate cancer-specific epitopes that are necessary to achieve a simultaneous activation of multiple T cell clones. To circumvent these issues, we generated separate SLP constructs covering the majority of HLA-I alleles and incorporated multiple PAP and PSMA HLA-I restricted epitopes along with an NY-ESO-derived longer peptide, which was only part of the research and preclinical analysis. Furthermore, HLA-II-restricted prostate cancer-specific epitopes were included in the TENDU vaccine constructs as strong and long-lived CD8+ T cell responses are dependent on CD4+ T cells.^38^

The generation of peptides for presentation on MHC-I molecules requires cleavage of proteins into peptides by immunoproteasomes so that peptides of the correct size enter the ER through transporter associated with antigen processing (TAP), where they are loaded onto MHC-I molecules.^39^ We therefore optimized the selected prostate-specific SLPs by adding a four-amino acid sequence in the N terminus of the HLA-I restricted prostate-specific epitopes to enhance the translocation of peptides through TAP using a TAP translocation-enhancing sequence (TTES). After a TAP translocation assay,^40^ five of the TTES-extended CD8+ epitopes were selected for further development based on cleavage and generation of the correct C terminus of the CD8+ epitopes as analyzed by MALDI-TOF mass spectrometry.

The next step in the development of the vaccination strategy was to analyze the antigen-specific T cell responses in an experimental system. In a clinical setting, patients might benefit from a tetanus vaccination before the initiation of the therapeutic conjugate vaccine cycle in order to increase anti-MTTE antibody levels. Additionally, tetanus vaccination can have beneficial effects on DC infiltration and activation at the vaccination site,^41^ so there is an opportunity for mutual benefits by smart clinical trial design. With this in mind, we vaccinated prostate cancer patients and healthy volunteers with a TTd-based vaccine (Boostrix). In this way, we could analyze the antigen-specific T cell responses in connection to the adjuvant effect of anti-MTTE binding sequences to antibodies in a physiological-like experimental system. Both prostate cancer patients and healthy individuals mounted an IgG1 response to MTTE. The IgG1 isotypes can bind to C1q, which is necessary for IC antigen presentation and cross-priming in *in vivo* models of intravenous injection of ICs.^42^^,^^43^

According to recall responses in blood samples from individuals before and after 2 weeks of vaccination with diphtheria, tetanus, and pertussis (DTP), one prostate cancer patient and one healthy individual responded with increased frequency of TNF-α+ memory CD8+ T cells after vaccination in the presence of LUR1-6 constructs, whereas IFN-γ expression was not detected in any of the individuals analyzed. The presence of CD8+ T cell clones specific for prostate cancer epitopes has been reported among healthy individuals and in early-stage disease, whereas IFN-γ expression in CD8+ T cells from advanced-stage prostate cancer patients tends to be very low or absent.^23^ In the blood loop system used here, the low frequency of cancer-specific T cell clones in the blood combined with the short time of re-stimulation may have been a reason for the limited sensitivity of the flow cytometry as a method of detection. In our previous study setup,^18^ the use of CMV+ donors and HLA-A∗0201 CMV pp65 tetramer led to a robust detection of antigen-specific T cells in the human whole blood loop system. However, in the current study, tetramers for characterization of HLA-restricted peptide-specific T cell clones were not available, limiting our capacity to detect the relevant T cell clones accounting for the poorer sensitivity. Another way to increase the sensitivity is to perform the assay on blood from patients who had expanded T cell clones reactive to the epitopes in the vaccine (via vaccination or through *ex vivo* expansion); this was, however, not performed. Exogenous addition of anti-MTTE together with the LUR1-6 constructs in whole blood also led to increased TNF-α release among memory CD8+ T cells in a prostate cancer patient not previously vaccinated with DTP.

In addition to the above, we also made use of transgenic humanized HLA-DR4 mice and assessed antibody and T cell responses after vaccination with LUG2, one of the peptide constructs that include an HLA-DR4 epitope. The transgenic HLA-DR4 mouse model has been extensively used to analyze immune responses to epitopes contained in pathogens, in autoimmune diseases, and to identify epitopes for cancer immunotherapy.^44^^,^^45^ Interestingly, we show that the HLA-DR4 animals seroconverted (anti-MTTE IgG measured) after the peptide conjugate vaccination and that they mounted a notable T cell response after vaccination with the construct, indicating that the CD4 epitope can aid in anti-MTTE-induced immune responses despite the mice not being seropositive prior to vaccination.

All different elements incorporated in TENDU have been analyzed for potential safety and toxicity issues. The MTTE sequence itself is part of the TTd, which has been widely used in global vaccination programs with minimal side effects. The SLPs contain PAP and PSMA CD4 and CD8 epitopes, which have been included in various formats in clinical trials and were well tolerated. In addition, *in silico* predictions have confirmed that the risk for off-target autoimmune triggered events by the peptide spacers incorporated into TENDU is low. To evaluate the preclinical safety of the constructs, we used a human *ex vivo* blood loop assay (ID.Flow) as well as a repeated toxicity study in rabbits. In the *ex vivo* blood model, we modeled the risk for immune-complex-mediated toxicity. The rabbit was chosen as the species for the *in vivo* study as they are known to respond well to immunization with tetanus vaccine, enabling the formation of immune complexes with tetanus antibodies and the LUG1-6 constructs. In addition, rabbits are prone to serum sickness,^46^ making them an excellent model for our studies. In both systems we evaluated safety with a focus on tetanus seropositive individuals, evaluating the risk for infusion reactions by assessing cytokine release and complement activation following exposure to a mixture of the clinical grade TENDU constructs (LUG1-6). No signs of broad cytokine release or complement activation were registered in either system at relevant doses of the construct. Importantly, peptide-peptide conjugate exposure was evident by a measurable increase in anti-tetanus antibody levels at the highest dose level, which was not observed in the non-vaccinated groups. This verifies that the exposure to LUG1-6 reached an exposure level that leads to an immune response in seropositive recipients. In rabbits, no clinical signs of toxicity were observed in any of the dosage groups. The subcutaneous injections did not induce any local adverse reactions, indicating that this route of administration is safe, and therefore could be used in the following clinical trial.

The Phase I study described herein is a first-in-human dose-selection study in medium to high-risk patients with relapse after primary radical prostatectomy, with the primary objective to investigate the safety and tolerability of the TENDU vaccine. For classical chemotherapy drugs, the purpose of phase I trials is essentially to determine the maximum tolerated dose. However, for immunotherapy, the anti-tumor effect may reach a plateau effect or even be less effective above a certain dose. For vaccine strategies, where usually none or low vaccine-related toxicity is observed, the optimal dose may be the minimum dose necessary to elicit an efficient immune response rather than the maximum tolerated dose. The biological effect of TENDU with regard to T cell activation in response to prostate cancer-specific epitopes cannot be studied in animal species. Thus, the dose levels in this Phase I trial were selected based on previous clinical experience with peptide-based cancer vaccines that share the goal (activation of T cell-specific responses) and/or the delivery mode (subcutaneous injection).^47^^,^^48^^,^^49^ A range of peptide concentrations has previously been used in various clinical trials for cancer immunotherapy. In a phase II clinical trial for castrate-resistant prostate cancer, doses of peptides up to 12mg (4 different peptides) with incomplete Freud’s adjuvant were used.^48^ In another study 25μg–900μg of an intracellular protein domain expressed by the tumor together with GM-CSF was administered.^49^ In a meta-analysis of cancer immunotherapy clinical trials from 1990 to 2011 including 68 clinical trials with more than 1300 patients, the range of the peptide doses used was between 10μg and 5000μg.^50^ In the same meta-analysis, 3% of the patients experienced grade 3/4 AEs, and 0.83% grade 3/4 systemic vaccine-related AEs. Among those peptide vaccine clinical trials that used a dose escalation strategy,^22^ none reported dose-limiting toxicity.^50^ However, dose escalation did not consistently correlate with increased cellular immune responses.

Based on the reported AEs in our Phase I study, the study treatment with the TENDU vaccine was safe and well tolerated, and no safety signals were raised.

As the study was a single-arm dose-finding Phase I study, clinical efficacy will be further investigated in future randomized clinical studies. However, our preliminary data showing T cell responses in five of six patients are promising. Interestingly, the most prominent T cell responses were noted in the patients with the highest measured anti-MTTE IgG levels after TENDU vaccination. We also observed a trend toward reduced levels of circulating tumor cells in the cohort receiving the highest vaccine dose and found that using a colocalization site for the preconditioning TTd-containing vaccine and TENDU was not successful despite being supported by literature.^41^ However, as the patients in cohort 3.2 did not display an increase in antibodies to TTd at visit 2 as for the other cohorts, it is plausible that the patients in cohort 3.2 had a more immunosuppressed status prior to inclusion than the patients in cohort 3.1. Alternatively, the co-administration protocol led to negative interference on specific immune responses against the epitopes in TENDU. Limitations of the current study pertain to the small number of patients in the clinical study along with limited efficacy data from both preclinical and clinical studies. In addition, there was no measured dose-limiting toxicity, indicating the need for further investigation into optimal dosing and larger cohorts. This would help to accurately assess whether immune-complex formation can break self-tolerance, thereby enhancing the immunogenicity of peptide vaccines and improving overall vaccine efficacy.

In summary, this study demonstrates the feasibility of producing a chemically synthesized peptide-peptide conjugate vaccine and assessing it in a clinical setting. The strategy showed no safety concerns during preclinical studies and, importantly, none in the clinical trial, where promising immune responses were observed. Consequently, we anticipate that optimizing the dosage, administration, and design will enable targeted vaccine drug delivery and cell activation, ultimately improving the immunogenicity of peptide vaccines.

## Materials and methods

### Synthesis of peptides and peptide constructs

All peptides and peptide constructs were produced at Leiden University Medical Center (LUMC). The core structure of the constructs was synthesized as previously described.^17^ Peptides were synthesized by Fmoc-based solid-phase chemistry.^51^ In short, the peptides were synthesized using solid-phase peptide synthesis on a TentagelS-Ac resin (Rapp, Tübingen) using a Syro II peptide synthesizer (MultiSyntech, Witten, Germany). Normal couplings (1.5 h–2 h) were performed using 9-fluorenyl methoxycarbonyl (Fmoc) amino acids carrying acid labile side-chain protection groups (were required). Activation of Fmoc amino acids was performed with PyBop and NMM, and azidopropionic acid was coupled using the succinimidyl ester. Fmoc deprotection was performed with 20 vol% piperidine in NMP. Washings were performed with NMP. Cleavage from the resin and side-chain deprotection was performed with Trifluoroacetic acid (TFA) containing 5% water and 2% triethylsilane. Purification was performed with rpHPLC. Analysis of the (purified) peptides was performed with UPLC-MS (Acquity, Waters, Milford, Massachusetts, USA) and MALDI-Tof MS (Bruker Microflex) and showed the expected molecular masses.

### Construct synthesis research grade

MTTE-SH (4.8 mg, 1.42 μmol) was dissolved in degassed H_2_O (800 μL) (Millipore) under argon atmosphere, the pH was adjusted to 6.0 by addition of NaHCO3 (0.5 M, 20 μL, degassed). To this solution, the core compound containing three maleimido and one cyclo-octyn group (0.234 mg, 0.24 μmol in 23 μL acetonitrile) was added as described previously.^17^ This mixture was stirred for 3 h after which mass spectrometry (Qtof) showed complete conversion of the core compound into the tris-MTTE-cyclo-octyn adduct. In addition, MTTE-SH and the related disulfide MTTE-S-S-MTTE were observed, resulting from the excess MTTE-SH that had been used. To this mixture was added azido-SLP (0.63mg, 0.289 μmol in 400 μL DMSO). In 1 h, the tris-MTTE-cyclo-octyn construct was converted into the final product as was monitored with rpHPLC analysis of the reaction mixture (UPLC Peptide BEH C18 Column, 300 Å C18, 1.7 μm, 2.1 × 100 mm, UPLC-MS, Acquity, Waters). The mixture was purified by rpHPLC on a Reprosil-Gold 200 Å C18, 5 μm, 250 × 10 mm, column (Dr. Maisch, Ammerbuch-Entringen, Germany) yielding 2.7 mg, 0.20 μmol construct, and 83% based on the core compound. The product was analyzed by mass spectrometry (Qtof) and the expected mass was observed. The MTTE peptide used to synthesize the constructs was: F-I-G-I-T-E-L-K-K-L-E-S-K-I-N-K-V-F-A-E-K-Y-A-R-V-R-A-K-C (MTTE-SH).

### Synthesis and purification of peptides under GMP conditions

All peptides were synthesized using a computer-controlled CS536 peptide synthesizer (CS Bio, San Carlos, CA USA, 1–4 mmol in a single run), or a computer-controlled Tribute synthesizer (PTI, Tucson, AZ, USA, 0.1–1 mmol in a single run). Solid-phase peptide synthesis according to the Fmoc-protocol was performed from the C terminus to the N terminus and commenced with a Tentagel-S-AC solid support for the epitope containing peptides or a Chemmatrix amide resin in case of the MTTE peptide. The synthesis of the peptide was performed by a process of repeating cycles in which the N-terminal Fmoc-group was cleaved before the addition of the next Fmoc-protected amino acid and coupling reagent. Various Fmoc-protected amino acids contain different protective groups in their side-chains. Conditions for the repetitive cleavage of the Fmoc-group were chosen such that neither the aforementioned C-terminal link to the solid support nor the protective groups of the side-chains were cleaved during this process.

The peptide was cleaved from the solid support with concomitant side-chain deprotection using a mix of TFA (95%) and water (5%). In the case of the azide peptides, cysteine (5g/100 mL cleavage mix) was added as a scavenger. The process was completed with a lyophilization step using a computer-controlled Steris Lyovac GT4 lyophilizer. The crude peptide was purified as described below.

Purification was performed using preparative gradient RPHPLC on a Prep4000 LC system (Waters, Milford, MA USA), a Waters 2487 dual λ UV-detector and an FCIII fraction collector. For each compound, a dedicated Dr. Maisch Reprosil-Pur C18-AQ 120 Å 10 μm (100 × 20 mm for the six azide peptides, 100 × 40 mm for the MTTE peptide) was used. The chromatographical system was controlled by Waters Empower software.

Analytical gradient RP-UPLC with UV/MS detection of the main fractions was performed using the following system: Waters Acquity UPLC system equipped with a photodiode array spectrophotometer and an SQD mass spectrometer with ESI probe and Empower software. The system uses a Waters BEH C18, 120 Å, 1.7 μm, 2.1 × 100 mm column.

### Synthesis and purification of the constructs (GMP)

Synthesis of the constructs was performed following a 1-pot three-step synthesis. In the first step, the MTTE peptide was coupled to the maleimide functionality of the TMCO core molecule via the side-chain of the N-terminal Cysteine residue. The reaction was performed at room temperature in a mixture of acetonitrile/water, pH ∼6.5. After completion of the reaction (approximately 30 min), the mixture was diluted (1:1) with 90% t-BuOH/water, and the pH was raised to 8.7 by the addition of 0.5M NaHCO3 to allow for ring opening of the three succinimide rings of the construct. The reaction was left at 30°C until analytical UPLC-MS analysis showed ring opening to be complete (approximately 6 days). Then the pH was lowered to 6.0 using a solution of 3% acetic acid in water and the azide-peptide was coupled to the cyclooctyn groups in the construct via a copperless click reaction.

The reaction mixture was stored at −20°C until purification.

Purification was performed using preparative gradient RPHPLC on a Prep4000 LC system (Waters, Milford, MA, USA), a Waters 2487 dual λ UV-detector, and an FCIII fraction collector. For each compound, a dedicated Dr. Maisch Reprosil-Gold C18 200 Å 5 μm 100 × 20 mm column was used. The chromatographical system was controlled by Waters Empower software.

Analytical gradient RP-UPLC with UV/MS detection of the main fractions was performed using the following system: Waters Acquity UPLC system equipped with a photodiode array spectrophotometer and an SQD mass spectrometer with ESI probe and Empower software. The system uses a Waters BEH C18, 300 Å, 1.7 μm, 2.1 × 100 mm column.

### Evaluation of the GMP constructs

Maleimide-thiol coupling to a core molecule was performed for MTTE-conjugation, and opening of the succinimide rings was performed to improve drug candidate stability. To achieve this, the pH was raised to 8.7 using 0.5M NaHCO3, and the reaction was left at 30°C until UPLC-MS analysis confirmed complete opening (approximately 6 days). Then the pH was lowered to 6.0 using a solution of 3% acetic acid in water and the azide-peptide was coupled to the cyclooctyn groups in the construct via a copperless click reaction. Confirmation of immunogenicity after ring opening was assessed by *in vitro* antigen presentation as described below.

#### *In vitro* antigen presentation assay

##### Cell lines

D1 cells are growth-factor dependent immature dendritic cells derived from C57BL/B6 mice (kindly provided by P. Ricciardi-Castagnoli, University of Milano- Bicocca, Milan, Italy). D1 cells were cultured as described in Winzler et al.,^52^ with the exception of replacing the supplement R1 supernatant with granulocyte-macrophage colony-stimulating factor (GM-CSF) (20 ng/mL). B3Z is a murine T cell hybridoma specific for the OVA-derived CD8+ epitope SIINFEKL in H-2Kb. B3Z cells were cultured in Iscove’s Modified Dulbecco’s Medium (IMDM) with 10% heat-inactivated FBS, 1% penicillin/streptavidin, and 50 μM 2-mercaptoethanol. The medium was supplemented with Hygromycin B (Invitrogen, Life Technologies, Rockville, MD) to select clones with a β-galactosidase construct under the NF-AT elements from the IL-2 promoter.^53^ The generation and culturing of hybridoma cell lines producing mouse anti-MTTE IgG1 and IgG2a antibodies were performed as previously described.^18^

The antigen presentation assay was performed as previously described.^17^ Briefly, immune complexes were pre-formed by incubating the antigen ([MTTE]_3_-SIINFEKL) with an antigen-specific antibody (anti-MTTE IgG1 or IgG2a) at 37°C for 30 min. The immune complexes were incubated with D1 cells (2.5 × 10^4^/well), which were subsequently incubated for another 24 h with B3Z cells (5 × 10^4^/well) at a DC:T cell ratio of 1:2. The immune complexes were pre-formed at 3-fold higher concentration and diluted once added to the D1 cells, concentrations shown in Figure 1D. The cells were lysed with a lysing solution (100 mM β-mercaptoethanol, 0.125% IGEPAL CA-630, 9 mM MgCl2) containing the β-galactosidase substrate chlorophenol red-β D-galactopyranoside (CPRG; 1.8 μg/mL) at 37°C for 6 h before the absorbance was measure at 595 nm using an iMark microplate reader (Bio-Rad).

### Selection of prostate cancer-derived epitopes

The Eurotransplant database (2013), based on HLA-typing of 4,000 donors from the Netherlands, Belgium, Germany, and Austria, was used to identify HLA class I alleles with high population coverage. CD8+ prostate cancer epitopes covering the selected HLA class I alleles were identified by a literature search in published papers and databases on prostate-specific epitopes.^21^^,^^22^^,^^23^ CD4+ prostate cancer epitopes were also identified and selected based on promiscuity and broad coverage of HLA-II restricted responses.^25^^,^^26^^,^^27^

### TAP and proteasome assays

Selected CD8+ and CD4+ prostate-specific epitopes were synthesized as 66 different SLPs, where each of the 11 CD8+ epitopes was combined with each of the six CD4+ epitopes. The SLPs also contained an N-terminal TTES sequence (see below). To determine if the SLPs were correctly cleaved by the immunoproteasome (creating the exact C terminus of the CD8+ epitope) we assessed the processing by MALDI-TOF: the SLPs were dissolved in DMSO (10 mg/mL). Each peptide (1 μL of the DMSO stock solution) was added to 300 μL aqueous buffer containing 0.5 μg immunoproteasome 20S (human, purified, BML-PW9645-0050, Enzo Life Sciences Farmingdale, NY, USA), 30 mM Tris-HCl (pH 7.2), 10 mM KCl, 5 mM MgCl2, and 1 mM DTT. The mixture was vortexed and incubated at 37°C for various time periods. At each time point (0 h and 24 h) an aliquot (50 μL) was taken from the digestion mixture, added to 4 μL formic acid, and then homogenized by vortexing, and stored at −20°C until analysis. For analysis, 1 μL of this solution was mixed with 1 μL matrix solution (10 mg/mL α-cyano-4-hydroxy cinnamic acid (ACH) in acetonitrile/water 1/1 containing 0.2% TFA) and spotted on a MALDI-TOF target plate. Samples at all time points were analyzed with MALDI-TOF mass spectrometry (Bruker Microflex or a Bruker Ultraflex instrument) revealing the proteasome-generated peptide fragments (>800 Da).

The SLPs that did not show a proteasome-generated CD8+ epitope after 24-h incubation were resynthesized with spacer sequences between the CD8+ and CD4+ epitopes, obtained using NetChop 3.1 (NetChop 3.1 - DTU Health Tech - Bioinformatic Services).^54^^,^^55^ Several spacer elements were applied in an attempt to improve the processing of the epitopes. This was performed by first predicting the cleavage of an SLP containing a spacer between CD8+ and CD4+ epitope (NetChop 3.1) and subsequently testing the expected improvement in a proteasome digestion experiment using MALDI-TOF mass spectrometry as described above. In this way it was confirmed that the known AAA sequence is a proper spacer for some combinations of epitopes (LUR2/LUG2 and LUR3/LUG3) in the SLPs and that the thus far unknown spacer QQQPPP is a useful spacer for C9H1 (LUR1/LUG1 construct).

In order to enhance ER translocation of the selected CD8+ epitopes, the TAREG prediction program (Immunomedicine Group: Tools >> Tapreg (ucm.es))^28^ was used to identify a TAP Translocation-Enhancing Sequence (TTES). The CD8+ epitopes were synthesized with the TTES at the N terminus, and the TAP translocation efficiency was analyzed and compared with that of the unmodified CD8+ epitope using TAP-expressing microvesicles and fluorescently labeled SLPs as described previously.^40^

### Evaluation of binding of GMP LUG1-6 constructs to human monoclonal anti-MTTE IgG1 antibody

Six peptide conjugates were selected for further evaluation: LUR1-6 and LUG1-6 (LUR and LUG are conjugates of the same peptide sequence with the difference of LUG being produced with GMP standard whereas LUR was produced for research purposes only).

An in-house ELISA was used to confirm antibody binding of the GMP-produced LUG1-6 constructs to a recombinant chimeric human monoclonal anti-MTTE IgG1 antibody as described below. ELISA plates were coated with 100 μL constructs diluted in Milli-Q water using a range of construct concentrations (0.000457-1 nmol/mL). The plates were covered and incubated at 4°C overnight. The plates were subsequently washed four times and blocked with 200 μL PBS containing 10% BSA/0.05% Tween 20 and incubated at room temperature (RT) for 1 h. After washing, the anti-MTTE IgG1 antibody, 0.1 μg/mL in PBS/1% BSA/0.05% Tween 20 was added. The plates were washed four times with 250 μL PBS/0.05% Tween 20 and the secondary antibody diluted 1:8,000 in PBS/1% BSA (anti-human kappa light chain secondary antibody) (Thermo Fisher #A18853) was added to all wells. After incubation for 1 h in RT in the dark, the plates were washed and 100 μL of TMB to the wells. The reaction was stopped with 100 μL 1M H2SO4 and the absorbance was acquired at 450–570 nm wavelength.

### Cohort description preclinical study

Eighteen patients from the prostate cancer urology department at Uppsala University Hospital and 13 patients from the oncology department were included to evaluate immune responses pre and post tetanus vaccination. The median age of the individuals from the cancer urology department was 70 years (range 61–86 years), whereas for the oncology department patients, the median was 75 years with a range of 60–85 years. Seven of the cancer patients from the oncology department had distant metastases at the time of sampling. Of these patients, 17 patients (combined from the urology and oncology departments) agreed to visit and do blood both pre and post-TTd vaccination.

Another cohort of age-matched male healthy volunteers (*n* = 5) and prostate cancer patients (*n* = 5) was recruited for the preclinical safety assessment and for the assessment of IgG and IgG1-antibodies to MTTE post-DTP vaccination. All individuals were vaccinated using the DTP-vaccine against tetanus, diphtheria, and polio Boostrix (GSK, Brendfort, UK). Table S3 shows patient characteristics and demographics for the preclinical study.

### MTTE antibody titers in human plasma

Anti-MTTE antibody titers in plasma from patients and healthy donors (pre- and post-DTP vaccination) were determined using an in-house ELISA. Streptavidin plates (Thermo Fisher Scientific, Waltham, MA, USA) were coated with in-house produced biotinylated MTTE peptide (including a spacer peptide sequence between the epitope and the biotin) overnight at 4°C. Sequence of the peptide: MTTE: FIGITELKKLESKINKVFSSSAFADVEAAZO-Biotin with the sequence SSSAFADVEAA providing a spacer element to give access to the MTTE epitope in the ELISA setup (Z, aminohexane acid, O, Lys (biotin)). The plates were washed with PBS (0.05% Tween 20) and blocked with PBS (10% BSA and 0.05% Tween 20) for 1 h at RT. The plasma was diluted in PBS (1% BSA and 0.05% Tween 20) and incubated for 2 h in RT. The MTTE-specific IgM and IgG antibodies were detected with secondary HRP-conjugated antibodies; rabbit anti-human IgG (polyclonal antibody from Dako; diluted 1:4,000), anti-IgG1 (Clone HP6070 from Thermo Fisher; diluted 1:500), anti-IgG4 (Clone HP6023 from Thermo Fisher; diluted 1:500), and anti-IgM (polyclonal from Dako, Glostrup, Denmark; diluted 1:1,000). The secondary horseradish peroxidase (HRP)-conjugated antibodies were diluted in PBS (1% BSA) and incubated on the plates for 1 h in RT. The reaction was developed with the substrate TMB (Dako) and stopped with 1 M H2SO4. The absorbance was read at 450–570 nm using an iMark microplate reader (Bio-Rad, Hercules, CA, USA). Data for Figures 2A–2D are plotted as absorbance (450–570 nm) values without background subtraction at dilution 1:10 for IgG1, IgG4, and IgM and at dilution 1:200 for IgG. Antibody titers in Figures 2E and 2F were determined based on how much the samples could be diluted before the antibodies could no longer be detected. A cutoff value for detection was set to absorbance level = 0.100.

### Blood loop assay

Whole blood was sampled in an open system and immediately mixed with heparin (Leo Pharma AB, Sweden) to a final concentration of 1 IU/mL. All materials in direct contact with whole blood were surface heparinized according to the manufacturer’s instructions (Corline AB, Sweden). The blood was, together with test materials (SLPs/peptide constructs at a final concentration of 125 nM of each of the six different peptide constructs/antibodies 40 μg/mL), applied to surface heparinized PVC tubings (Corline AB, Uppsala, Sweden) that were sealed with specialized metal connectors and thereby forming loops. The blood-filled loops were rotated on a wheel at 37° and at different time points aliquots were acquired for plasma and cellular analysis. The blood aliquots were mixed with EDTA (10 mM) to stop reactions after acquisition from the loops. For intracellular staining of cytokines, brefeldin A was added after 2 h of rotation followed by another 4 h of rotation before flow cytometry analysis as described below. The automated hematology analyzer XP-300 (Sysmex, Kobe, Hyogo, Japan) analyzed the blood for platelets and white blood cell counts, etc., and was used to assess the blood platelet count.

### Flow cytometry analysis

Antibodies for flow cytometry analysis were purchased from Biolegend (San Diego, CA, USA): anti-CD3 (Clone UCHT1), anti-CD4 (Clone OKT-4), anti-CD8 (clone SK1), anti-CD45RO (Clone UCHL1), anti-IFN-γ (Clone 4S.B3), and anti-TNF-α (Clone MAb11). Whole blood was stained with cell surface-specific antibodies before RBC lysis using FACS lysing solution (BD Biosciences, Franklin Lakes, NJ, USA) according to the manufacturer’s instructions. The cells were washed and fixed with BD cytofix/cytoperm buffer at 4°C in the dark for 20 min. To permeabilize the cells, they were first washed and then incubated with Perm/Wash Buffer (BD Biosciences) in RT for 10 min. The cells were stained for intracellular cytokines (IFN-γ and TNF-α) for 30 min at 4°C in the dark. The cells were washed in PBS with 1% BSA and 3 mM EDTA (Sigma-Aldrich, St. Louis, MO, USA), and analyzed using Canto II flow cytometer (BD Biosciences) or Cytoflex (Beckman Coulter, Brea, CA, USA). The cell populations were gated and analyzed using FlowJo (Tree Star).

### Evaluation of epitope-specific T cell responses in humanized HLA-DR4 mice

Female HLA-DR4 transgenic mice on a C57/Bl6 background^56^ (12 weeks at the start of the study) were purchased from Taconic (Germantown, MD, USA). A group of mice was vaccinated with a LUG2 construct (20 μg) by subcutaneous administration at the tail base followed by a boost 2 weeks later. A week later, the mice were euthanized, and the spleens were collected for the generation of single-cell suspensions for analysis with ELISPOT as described below. A separate group of matched HLA-DR4 mice was used as controls for the analysis of anti-MTTE titers before LUG-2 administration.

For detection of mouse anti-MTTE immunoglobulins in the HLA-DR4 mice, biotinylated MTTE or a scrambled version of ETTM were coated overnight on streptavidin-coated plates (Thermo Fisher Scientific). After washing with PBS, 0.05% Tween, and 20.1% BSA, the plates were blocked with PBS, 10% BSA, and 0.05% Tween 20 for 1 h at RT. The mouse serum was diluted with PBS, 1% BSA, and 0.05% Tween 20 and incubated in the plate for 2 h. The mouse anti-MTTE Ig was detected using HRP-conjugated anti-mouse Ig polyclonal antibody (PO447) (Dako, Denmark). The TMB substrate (Thermo Fisher Scientific) was used and the reaction was stopped with 1M H2SO4. Optical absorbance (OD) acquired at 450 nm after subtraction of 570 nm is presented.

#### ELISpot for evaluation of IFN-γ producing T cells in response to SLPs contained in the LUG2 constructs

The immunogenicity of the SLP contained in LUG2 construct with and without the TAP sequence: ARWWLLHETDSAVAAARQIYVAAFTVQAAAE (UV02) and LLHETDSAVAAARQIYVAAFTVQAAAE (UV08) were determined using the splenocytes harvested from immunized mice in an *ex vivo* IFN-γ ELISpot assay (ELISpot kit for mouse IFN-γ/3321-2A, Mabtech, Stockholm, Sweden) according to the instructions from the manufacturer. Briefly, 0.5 × 10^6^ freshly isolated splenocytes per well were seeded in triplicates into the pre-coated IFNγ ELISpot plate along with 10 μg/mL of the respective SLPs. The cells were then incubated at 37°C in a 5% CO_2_ incubator for 48 h. Subsequently, plates were washed with PBS and a biotinylated detection antibody against mouse IFN-γ was added. Plates were incubated at RT for 3 h followed by washing and addition of streptavidin alkaline phosphatase. After incubation (2 h at RT) and washing, a development solution (BCIP/NBT, Bio-Rad) was added. Once spots developed, the reaction was stopped by rinsing the plates under tap water. Plates were then left to dry and the spots were quantified using an ELISpot plate reader (Cellular Technology Limited, Shaker Heights, OH, USA). SEB, the staphylococcal enterotoxin-B, at (2.5 μg/mL) was used as a positive control, and unstimulated splenocytes (cells alone) were used as a negative control for every ELISpot assay.

### Cytokine and complement analysis in plasma from Boostrix-vaccinated prostate cancer patients and healthy controls

Evaluation of risk for infusion reactions (cytokine release syndrome) was done by measuring cytokine/complement activation in the blood. Approximately 2 weeks after vaccination with Boostrix (Tetanus Toxoid-, Reduced Diphtheria Toxoid-, and Acellular Pertussis Vaccine) blood was collected from five healthy individuals and five prostate cancer patients. For this evaluation, two samples from the urology department and three samples from the oncology department were used.

The blood was treated with three different concentrations of the TENDU vaccine mixed constructs LUG1-6 using 0.05 μg/mL, 0.5 μg/mL, and 2.5 μg/mL from each individual construct. Alemtuzumab (3 μg/mL) was used as a positive control for cytokine release and complement activation. Plasma harvested after 0 and 4 h in the blood loop assay was used for concentration determination of IFN-γ, IL-1β, IL-2, IL-6, IL-8, IL-10, and TNF-α using Mesoscale V-plex kit (MSD Discovery, Kenilworth, NJ, USA) according to the manufacturer’s instructions. The lower limit of detection (LLOD) was calculated by MSD software and defined as 2.5x SD above the zero calibrator. The upper limit of detection (ULOD) is calculated by MSD software from the signal value of the Standard-1. Lower and upper limits of quantifications (LLOQ and ULOQ) are verified by MSD and calculated from the standard curve and percentage recovery of diluent standards with the precision of 20% and accuracy 80–120%.

Plasma harvested after 0 and 15 min in the blood loop assay was analyzed for complement activation (C3a and C5a) with ELISA kits from Hycult Biotech (Uden, Netherlands) according to the manufacturer’s instructions.

### *In vivo* toxicology

Toxicity testing in rabbits was performed by Meditox (Konárovice, Czech Republic). The toxicity of the TENDU conjugates (LUG1-6) was analyzed in TTd seropositive and seronegative rabbits. In this study, 31 male rabbits were vaccinated with a TTd vaccine and were allocated in five different groups based on the titers. The G1 is a vehicle group, G2–4 are seropositive animals that were then dosed with LUG1-6 at three doses (6 × 10 μg, 6 × 100 μg, and 6 × 240 μg), and the G5 is a seronegative group dosed with the highest dose of LUG1-6. LUG1-6 was then given at weeks 9, 11, 13, and 15 and titers were again analyzed on week 15. The following parameters were evaluated: clinical signs including body temperature, body weight, and food consumption; visual inspection of the injection sites during the in-life phase; hematology; clinical chemistry with weekly blood sampling for analysis of urea and creatinine to evaluate possible kidney toxicity (due to deposition of immune complex in the kidney blood vessels); urine analysis; full histopathology; analysis of cytokine release in connection with the first and last dosing occasion; and tetanus titers before the start and at the end of the study.

Blood samples were collected in K3 EDTA tubes on week 15 before TENDU administration and post-TENDU administration at 4 h and 24 h. Blood samples were centrifuged (3,500 rpm for 10 min, at 4°C). The plasma was collected and stored at −20°C until analysis with ELISA. For MTTE-titer determination, blood samples for serum preparation were collected on week 8, 1 week after the last tetanus vaccination and before TENDU administration, and on week 15 after the last administration of TENDU or vehicle. The blood was collected in TEPVAL tubes without anti-coagulant, centrifuged at 6,000 rpm for 10 min, and the serum was subsequently frozen at ≤20°C until analysis.

#### ELISA for cytokine detection in rabbit plasma

The following ELISA kits were used for analysis of cytokines in rabbit plasma: RayBio Rabbit IL-8 cat. no ELL-IL-8-1, RayBio IL-1β cat. no ELL-IL1b-1, and RayBio Rabbit IFN-γ cat. no ELL- IFN-γ −1 (Norcross, GA, USA). Cytokine analysis was performed according to the manufacturer’s instructions.

#### ELISA for detection of anti-MTTE titers in rabbit serum

Determination of anti-MTTE titers in rabbit serum from male rabbits pre-vaccinated with TTd, was performed using an in-house ELISA. As a control, rabbits without pre-vaccination with TTd were included. Briefly, biotinylated MTTE peptides (1 μg/mL) were coated on streptavidin-coated NUNC immune MaxiSorp plates and incubated at 40°C overnight. After washing, the serum was added to the plates at different dilutions. Detection of anti-MTTE antibodies was performed using goat anti-rabbit IgG antibody conjugated to alkaline phosphatase (1:5,000). The substrate 4-nitrophenyl phosphate disodium salt hexahydrate (1 mg/mL) was added to the plate and the absorbance was acquired at 405 nm. The titer is the dilution value when 50% of the coated peptides are bound to a primary antibody in relation to the maximum antibody binding. A recombinantly produced chimeric anti-MTTE IgG1 antibody served as standard, and a 5 PL standard curve was used for the determination of anti-MTTE IgG serum concentration.

### Clinical trial assessment of safety of the peptide-peptide conjugate vaccine approach

To assess the safety profile of the peptide-peptide conjugate in a clinical context, a Phase I clinical trial was designed employing the conventional 3 + 3 dosing escalation approach. The target patient population was high-risk prostate cancer patients with documented progressive disease (radiologically assessed, PSA progression, or both) after radical prostatectomy eligible for salvage radiotherapy and short-term (6 months) ADT. The patients were not selected based on HLA profile. Patient characteristics and demographics for the clinical trial are presented in Table S4. The primary objective of this trial was to evaluate safety. Secondary endpoints encompassed immune responses and preliminary anti-tumor responses. Four out of the six TENDU conjugates were selected to be used for the clinical assessment, based on the availability of the small-scale produced GMP material (LUG1, LUG3, LUG4, and LUG5). All patients taking part in the study were vaccinated with a Boostrix vaccine (including TTd) 7–10 days prior to the first TENDU treatment. The Boostrix vaccine injections were administered intramuscularly, in accordance with the summary of product characteristics (SmPC).

Three different doses of the TENDU vaccine were investigated: 40 μg, 400 μg, and 960 μg. The vaccine was administered four times in total during the 42-day (6 weeks) treatment period: on day 1, day 14, day 28, and day 42 (visit 2 to visit 5). After the TENDU vaccination, each patient was followed up at the study site for at least 6 h. Blood sampling took place before treatment (0 h) and at 15 min, 1 h, and 4 h post-TENDU vaccination.

Patients were included in the study according to a dose escalation 3 + 3 design. The first three patients included (cohort 1) received the lowest dose of the TENDU vaccine (40 μg), the next three patients (cohort 2) received the medium dose (400 μg), the next three patients (cohort 3.1) received the highest dose (960 μg). In cohorts 1, 2, and 3.1, the vaccine was administered in the abdomen. The last three patients (cohort 3.2) received the highest dose (960 μg) administered in the same arm as the Boostrix injection. After treatment of the first patient in each cohort was completed (four doses administered on day 1, day 14, day 28, and day 42), a safety review committee (SRC) evaluated all available data collected from baseline to the fourth TENDU vaccination. If no safety concerns were identified, patients 2 and 3 in each dose cohort could be included in the study. Safety evaluations were also conducted after each cohort was completed. Safety assessments primarily focused on the occurrence of vaccine-related toxicities classified as Grade 3–4 (dose-limiting toxicities [DLTs]) and those that were deemed possibly, probably, or definitely related to the TENDU vaccine, excluding local reactions and general constitutional symptoms.

The additional high-dose group (3.2) was designed to assess if administration of TTd pre-conditioned lymph node can impact immune responses to the subsequent administration of TENDU based on a published work by Mitchell et al.^41^

#### Cytokine analyses

Cytokine analyses (INF-γ, IL-1β, IL-2, IL-4, IL-6, IL-8, IL-10, IL-12p70, IL-13, and TNF-β) were performed using a Mesoscale V-plex proinflammatory panel 1 human kit (MSD Discovery, Kenilworth, NJ, USA) as described above. Complement activation (C3a and C5a) was assessed using ELISA kits from Hycult Biotech (Uden, Netherlands) according to the manufacturer’s instructions.

#### Anti-tetanus and anti-MTTE-levels in blood

Anti-MTTE antibody titers in plasma from patients and healthy donors (pre- and post-DTP vaccination) were determined using an in-house ELISA as described above.

#### Assessment of T cell responses

PBMCs from whole blood samples were isolated and cryopreserved until analysis. PBMCs were thawed, plated in 48-well plates (6 M cells/mL), and stimulated with peptides of the SLPs of the TENDU vaccine (10 μM) and poly-ICLC (20 μg/mL) (Oncovir) and cultured for 12 days. Cells were supplemented with IL-2 (20 IU/mL), on four occasions during culturing. The cells were reseeded in triplicate in an IFN-g release ELISpot assay setup plate and stimulated with peptides covering CD4 or CD8 epitopes of the SLPs for 22 h. The IFN-g-secreting cells were evaluated using IFNg ELISpot (CTL immunospot, HIFNGP-2M/5) according to the manufacturer’s instructions.

A positive immune response was considered when the mean spot count from stimulated cells exceeded the number of mean counts from the unstimulated cells plus 2x standard deviation of the triplicates. The spot count of the stimulated cells minus the spot count of unstimulated cells per 1 M cells is presented in the figures.

#### Preliminary anti-tumor response

The GILUPI CellCollector was used to harvest circulating tumor cells. The CellCollector is a medical wire coated with anti-EpCAM antibodies. The cell collector was inserted into the cubital vein for 30 min. At this point, the wire was extracted, washed in PBS, and the captured cells were collected and stained with Hoechts 33342 (Sigma), rabbit anti-PDL1 (Cell Signaling), mouse anti-PSMA conjugated with Alexa 488 (Novus Biologicals), and mouse anti-PAP (Thermo Fischer Scientific) antibodies. Secondary antibodies were goat-anti-mouse Alexa 647 or goat-anti-rabbit Alexa 555 (both from Thermo Fischer Scientific). The images were examined under a fluorescence microscope (Zeiss Axio Imager A1m from Carl Zeiss Microscopy GmbH, Göttingen, Germany).

A positive finding was defined with the following criteria: Intact morphology with a cell diameter ≥4 μm; core staining (Hoechst) must be positive and distinguishable from other markers; and cells must be positive for at least PAP, PDL1, or PSMA. Additionally, for individual markers (PAP, PDL1, and PSMA), a cutoff of a minimum three cells was used for cells to be deemed positive. If there were fewer than three stained cells, the value was set to 0.

### Ethical permissions

The local ethical committee approved the blood sampling and diphtheria, tetanus, and pertussis (DTP) vaccination of prostate cancer patients and healthy volunteers (Ethical Permission 2015-430). Routine personnel at Clinical Trial Consultants (CTC) performed the DTP vaccinations with a standard vaccine mixture.

#### Mouse studies

Animal use and care were in accordance with EU Directive 2010/63/EU and UK Home Office Code of Practice for the housing and care of animals bred, supplied, or used for scientific purposes. The studies were undertaken with the UK Home Office approval.

#### Rabbit studies

The study design was approved by the Institutional Animal Care and Use Committee (IACUC) and the Committee for Animal Protection of the Ministry of Health of the Czech Republic (75/2016). Procedures used in this study were designed to conform to accepted practices and to minimize or avoid causing pain, distress, or discomfort to the animals. The number of animals selected for use in this study was considered to be the minimum number necessary to meet scientific and regulatory guidelines for this type of study.

#### TENDU clinical trial

All information about the clinical study, including the patient information and the IC form, was prepared and used for the protection of the human rights of the patient, according to ICH GCP guidelines and the Declaration of Helsinki. Signed IC forms were obtained from each patient participating in this study after an adequate explanation of the aims, methods, objectives, and potential hazards of the study and prior to undertaking any study-related procedures. The study was approved by the local research ethics committee (ethical permission 175592).

### Statistics

Statistical analyses were performed using GraphPad Prism version 8.01 (GraphPad Software, San Diego, CA, USA). Statistical analyses for significances in the levels of MTTE antibodies, cytokine production, and complement activation in the blood loop system were performed with Wilcoxon matched-pairs test (nonparametric) or paired Student’s t test on Log10 transformed values above LLOQ with Holm-Sidak correction for multiple comparisons; ∗<0.05; ∗∗<0.01, ∗∗∗*p* < 0.001. Values above ULOQ were set as ULOQ in the statistical analysis. Zero values are not presented due to the logarithmic scale. Values below LLOQ were set as LLOQ in the statistical analysis. Analysis of differences in the cytokines concentrations in rabbit plasma was performed using a repeated measures two-way ANOVA with Tukey post hoc test.

## Data availability

The datasets generated and/or analyzed during the current study are available from the corresponding author on reasonable request.

## Acknowledgments

The work was supported by funding from 10.13039/501100003246NWO to J.W.D. and from BIOX/Vinnova to S.M. The preclinical and clinical work was sponsored by Immuneed AB and Ultimovacs ASA/AB. The vaccine platform involves a patent family that is licensed to Ultimovacs AB from LUMC involving the authors with affiliations to these institutes.

## Author contributions

Conceptualization: J.W.D. and S.M. Data curation: E.F., R.A.C., A.N., G.T., A.R.P.M.V., A.B., M.W., F.L., I.D., M.Lo., N.B., J.J.N., N.D., C.L.M.C.F., S.M., M.A., S.J., F.A.O., M.H., M.La., G.U., S.L., J.L.J., W.L., J.W.D., and S.M.M. Formal analysis: E.F., R.A.C., A.N., G.T., A.R.P.M.V., A.B., M.W., F.L., I.D., M.Lo., N.B., J.J.N., N.D., C.L.M.C.F., S.M., M.A., S.J., F.A.O., M.H., M.La., G.U., S.L., J.L.J., W.L., J.W.D., and S.M.M. Funding acquisition: J.W.D. and S.M.M. Investigation: E.F., R.A.C., A.N., G.T., A.R.P.M.V., A.B., M.W., F.L., I.D., M.Lo., N.B., J.J.N., N.D., C.L.M.C.F., S.M., M.A., S.J., F.A.O., M.H., M.L., G.U., S.L., J.L.J., W.L., J.W.D., and S.M.M. Methodology: E.F., R.A.C., A.N., G.T., A.R.P.M.V., A.B., M.W., F.L., I.D., M.Lo., N.B., J.J.N., N.D., C.L.M.C.F., S.M., M.A., S.J., F.A.O., M.H., M.La., G.U., S.L., J.L.J., W.L., J.W.D., and S.M.M. Project administration: E.F., R.A.C., A.N., G.T., A.R.P.M.V., A.B., M.W., N.B., J.J.N., N.D., C.L.M.C.F., S.M., M.A., S.J., F.A.O., G.U., S.L., J.L.J., W.L., J.W.D., and S.M.M. Resources: E.F., R.A.C., A.N., G.T., A.R.P.M.V., A.B., M.W., F.L., I.D., M.Lo., N.B., J.J.N., N.D., C.L.M.C.F., S.M., M.A., S.J., F.A.O., M.H., M.La., G.U., S.L., J.L.J., W.L., J.W.D., and S.M.M. Software: E.F., R.A.C., G.U., J.W.D., and S.M.M. Supervision: F.A.O., M.H., G.U., S.L., J.L.J., W.L., J.W.D., and S.M.M. Validation: E.F., R.A.C., A.N., G.T., J.L.J., W.L., J.W.D., and S.M.M. Visualization: E.F., R.A.C., A.N., A.B., M.W., F.L., I.D., M.Lo., M.La., G.U., S.L., J.L.J., W.L., J.W.D., and S.M.M. Writing – original draft: E.F., R.A.C., A.N., M.Lo., M.La., J.W.D., and S.M.M. Writing – review & editing: E.F., R.A.C., A.N., G.T., A.R.P.M.V., A.B., M.W., F.L., I.D., M.Lo., N.B., J.J.N., N.D., C.L.M.C.F., S.M., M.A., S.J., F.A.O., M.H., M.La., G.U., S.L., J.L.J., W.L., J.W.D., and S.M.M.

## Declaration of interests

The individual affiliations stated describe the potential conflicts of interest to the industrial parties involved. I.D. holds a current position with AstraZeneca but the work was performed under a separate affiliation and AstraZeneca has no connection to the work presented herein. W.L. has a consultant agreement with Ultimovacs ASA. We have registered patents and patent applications related to this work.

## Supplemental information

Document S1. Figures S1–S4 and Tables S1–S4

Document S2. Article plus supplemental information
