# Supplementary material for: Preclinical development and clinical safety assessment of a synthetic peptide conjugate enabling endogenous antibody binding to promote innate receptor engagement
Source: Mol Ther Oncol. 2025 Feb 20;33(2):200954. doi: 10.1016/j.omton.2025.200954 (PMC12166800; doi:10.1016/j.omton.2025.200954)
Supplement: Document S1. Figures S1–S4 and Tables S1–S4 [file mmc1.pdf]

OMTON, Volume *33*

Supplemental information

Preclinical development and clinical safety assessment of a synthetic peptide conjugate enabling endogenous antibody binding to promote innate receptor engagement

Erika A.K. Fletcher, Robert A. Cordfunke, Aikaterini Nasi, Gunilla Törnqvist, Rob R.P.M. Valentijn, Anna Bergqvist, Marita Westhrin, Wenche Rasch, Frida Lindqvist, Inken Dillmann, Martin Lord, Neanke Bouwman, Jacques J. Neefjes, Kees L.M.C. Franken, Stephanie McArdle, Murrium Ahmad, Silvia Johansson, Ferry Ossendorp, Michael Haggman, Maria Lampinen, Gustav Ullenhag, Sam Ladjevardi, Justyna Leja-Jarblad, Wolfgang Lilleby, Jan Wouter Drijfhout, and Sara M. Mangsbo

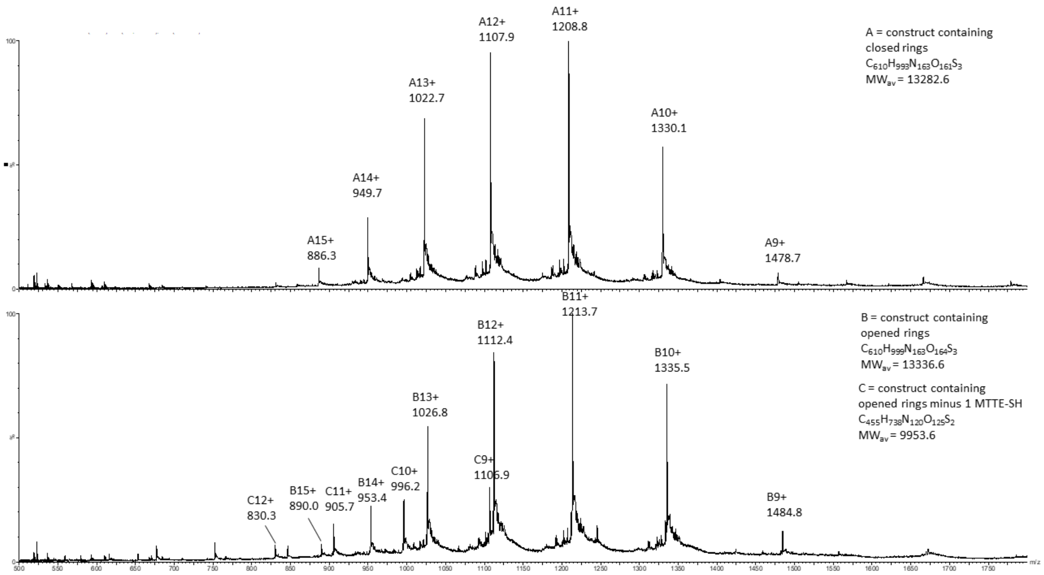

Figure S1. Stability of constructs with succinimide rings under basic conditions

Q-TOF spectra of constructs at 0h and 46h in 50 µl of buffer pH 8.7 (55 mM NaHCO3 pH 8.7, 11 % MeCN and 40 % tert-Butanol), incubated at 30°C. Upper part of the figure: the multiple charged ion spectrum of the sample at t=0 hours. Lower part of the figure: the multiple charged ion spectrum of the sample at 46 hours. If one succinimide ring is opened by hydrolysis the additional mass is 18 Da. The construct contains three succinimide rings, and if they all three opened by hydrolysis the additional mass is 54 da. If part of the construct with opened rings loses one MTTE-SH, the loss in mass is 3383 Da. As shown in the lower side of the figure, within 46 hours virtually all succinimide rings open and part of the molecules loose MTTE-SH.

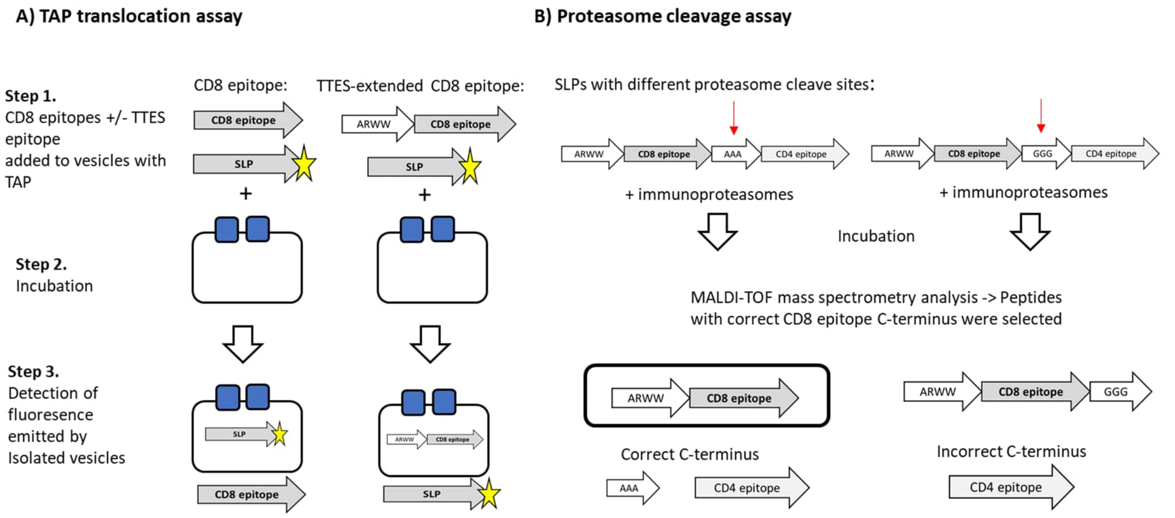

Figure S2. Selection and optimization of epitopes and SLPs based on TAP translocation efficiency and proteasome cleavage.

The TAP translocation efficiency of TTES extended CD8+ T cell epitopes was analyzed using TAP expressing microvesicles and fluorescently labelled SLPs. CD8 epitopes, with and without the TTES-extension, were titrated in to compete out the internalization of the SLP and thereby reduce the fluorescence of the vesicles (A). The selected SLPs were tested using immunoproteasomes for generation of correct C-terminus of CD8 epitopes with MALDI-TOF. Spacer sequences were introduced to some of the SLPs for generation of correct C-terminus (B).

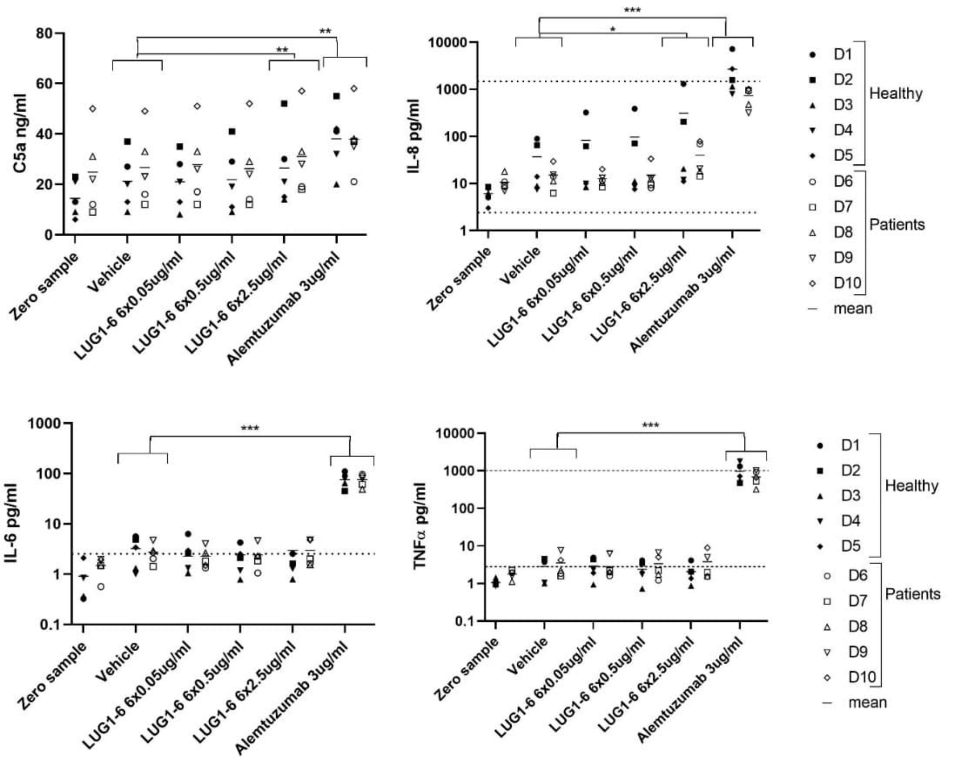

Figure S3. Analysis of TENDU toxicity in a human blood loop assay

Concentrations of C5a, IL-8, IL-6 and TNF-α. Calculated mean concentration values for C5a and raw mean concentrations of IL-8 IL-6 and TNF-α are presented. Plasma samples were collected from the whole blood loop assay at 0 (zero) and 4 hrs for cytokines (analysed using an MSD assay) and 0 time point and 15min for C5a (measured by ELISA). Vehicle is a negative control, while alemtuzumab (3 µg/ml) is a positive control. The mean of each group is indicated with a horizontal line. Calculated LLOQ and ULOQ are marked with dotted lines. n=5 (healthy and patients). Paired Student's t-test done on Log10 transformed values above LLOQ with Holm-Sidak correction for multiple comparisons) * $0.01<p<0.05$; ** $0.001<p<0.01$; *** $p<0.001$. Values above ULOQ were set as ULOQ in the statistical analysis.

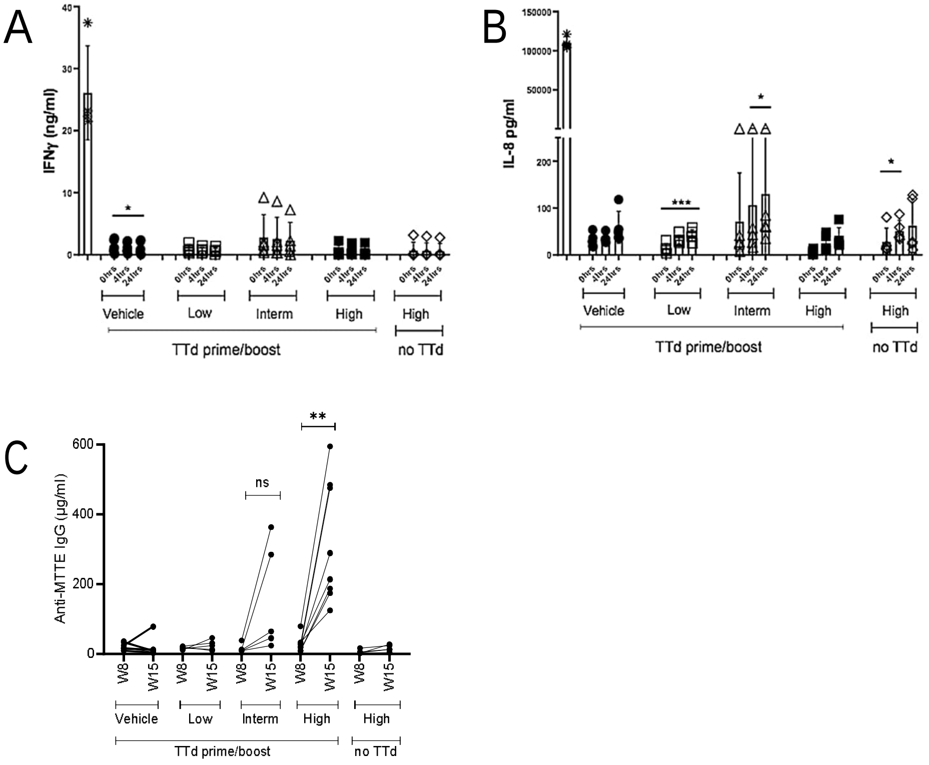

Figure S4. Rabbit IFN-**γ**, IL-8 and anti-MTTE concentrations pre- and post- TENDU vaccination

Plasma concentrations of (A) IFN-γ and (B) IL-8 in TTd vaccinated rabbits. Blood was collected before (0 hrs) and 4h and 24h after the 4th LUG1-6 administration (week 15). The isolated plasma was analysed using rabbit ELISA kits from Raybiotec. Replicates with Calibrator #8 (n=2) were used for LOD and LOQ calculations. The white bar represents positive control (rabbit plasma spiked with a known amount of IFN-γ or IL-8). The LOD and LOQ for IFN-γ was 0.32 ng/ml and 0.63 ng/ml respectively. The LOD and LOQ for IL-8 was 0.85 pg/ml and 1.73 pg/ml respectively. (C) Determination of anti-MTTE concentrations in serum from male rabbits pre-vaccinated with TTd, and subsequently vaccinated four times with low, intermediate or high dose of TENDU performed using an in-house ELISA. As a control, rabbits without pre-vaccination with TTd were included. Absorbance was acquired at 405nm. The results were analysed using a repeated measure two-way Anova with Tukey's post-hoc test, $p<0.05$*, ** $p<0.01$, $p<0.001$***, ns=non-significant.

Table S1. A selection of the 66 created SLPs after proteasome cleavage, assessed by MALDI-TOF.

Allele	Code	Sequence	Proteasome processing	Selected for vaccine
A1	C4 H1	A R W W G M P E G D L V Y G Q D L F G I W S K V Y D P L	+	
A1	C4 H2	A R W W G M P E G D L V Y T E D T M T K L R E L S E L S	-	
A1	C4 H3	A R W W G M P E G D L V Y G K V F R G N K V K N A Q L A	+	
A1	C4 H4	A R W W G M P E G D L V Y T G N F S T Q K V K M H I H S	+	Yes (LUG4)
A2	C5 H6	A R W W L L H E T D S A V A T A R R Q I Y V A A F T V Q A A A E	+	
A2	C5 H6	A R W W L L H E T D S A V A A A A R Q I Y V A A F T V Q A A A E	-	
A2	C5 H6	A R W W L L H E T D S A V A A A R Q I Y V A A F T V Q A A A E	+	Yes (LUG2
A2	C11 H2	A R W W S L S L G F L F L A A A T E D T M T K L R E L S E L S	+	
A2	C11 H3	A R W W S L S L G F L F L A A A G K V F R G N K V K N A Q L A	+	Yes (LUG3)
A2	C11 H4	A R W W S L S L G F L F L A A A T G N F S T Q K V K M H I H S	+	
A3,A11,A31,A33	C8 H4	A R W W K V F R G N K V K T G N F S T Q K V K M H I H S	+	
A3,A11,A31,A33	C8 H5	A R W W K V F R G N K V K N Y T L R V D C T P L M Y S L	+	Yes (LUG5)
A24	C9 H6	A R W W N Y A R T E D F F Q Q Q P P P P R Q I Y V A A F T V Q A A A E	+	
A24	C9 H1	A R W W N Y A R T E D F F Q Q Q P P P G Q D L F G I W S K V Y D P L	+	Yes (LUG1)
A24	C9 H2	A R W W N Y A R T E D F F Q Q Q P P P T E D T M T K L R E L S E L S	+	
A24	C3 H5	A R W W L Y C E S V H N F N Y T L R V D C T P L M Y S L	-	

Table S2. TAP translocation efficiency of selected CD8 epitopes with and without TTES extension.

Code#	CD8 epitope sequence	TTES-extended CD8 epitope sequence*	TAP translocation of CD8 epitope sequence	TAP translocation of TTES-extended peptide
1	ILLWQPIPV	ARWWILLWQPIPV	Insoluble	Insoluble
2	YLPFRNCPR	ARWWYLPFRNCPR	Bad	Good
3	LYCESVHNF	ARWWLYCESVHNF	Average	Bad
4	GMPEGDLVY	ARWWGMPEGDLVY	Bad	Good
5	LLHETDSAV	ARWWLLHETDSAV	Average	Good
6	MMNDQLMFL	ARWWMMNDQLMFL	Insoluble	Insoluble
7	VLAGGFFLL	ARWWVLAGGFFLL	Insoluble	Insoluble
8	KVFRGNKVK	ARWWKVFRGNKVK	Bad	Good
9	NYARTEDFF	ARWWNYARTEDFF	Average	Good
10	LLAVTSIPSV	ARWWLLAVTSIPSV	n.t.	n.t.
11	SLSLGFLFL	ARWWSLSLGFLFL	n.t.	n.t.

Table S3. Patient characteristics and demographics, preclinical study

**Immune responses pre-and post DTP-vaccination**	Urology unit	Oncology unit
n	18	13
Age, median (IQR)	70 (68-74)	75 (71-79)
PSA, median (IQR)	7.9 (5.4-21.3)	0.1 (0.05-1.8)*
Grade**, median (IQR)	3 (2-4.3)	4 (2-5)
**Preclinical safety assessment**		
n	2	3
Age, median (IQR)	71 (70-72)	73 (67-78)
PSA, median (IQR)	5.8 (3.4-8.1)	0.05 (0.05-5.8)
Grade**, median (IQR)	7(7-7)	9 (7-9)

*One sample lacking value, lower limit of detection set to absolute value
**Grade=prognostic grade group based on gleason score

Table S4. Patient characteristics and demographics, clinical trial

	Cohort 1 TENDU vaccine 40µg (n=3)	Cohort 2 TENDU vaccine 400µg (n=3)	Cohort 3:1 TENDU vaccine 960 µg Administered in abdomen (n=3)	Cohort 3:2 TENDU vaccine 960 µg Administered in arm (n=3)	Total (n=12)
Age (years), mean (SD)	68.0 (7.2)	64.7 (5.5)	69.0 (7.0)	63.0 (7.0)	66.2 (6.3)
Radical prostatectomy, yes	3 (100%)	3 (100%)	3 (100%)	3 (100%)	12 (100%)
ECOG performance status screening (ECOG) 0*	3 (100%)	3 (100%)	3 (100%)	3 (100%)	12 (100%)
PAP and PSA at screening					
PAP (ng/L) Mean (SD)	510 (140)	416 (124)	649 (223)	628 (167)	551 (173)
PSA (ug/L) Mean (SD)	0.28 (0.13)	0.55 (0.22)	0.34 (0.11)	0.28 (0.02)	0.36 (0.17)
ISUP grade**	GG4	GG5	GG4	GG3	
	GG5	GG4	GG4	GG3	
	GG5	GG5	GG4	GG5	
N-status (pN) 1***	2 (67%)	1 (33%)	0 (0%)	0 (0%)	
PSMA-PET/CT Scan Screening positive	1 (33%)	1 (33%)	0	1 (33%)	3 25%)
